# Eukaryotic metagenome-assembled genomes recovered from deep metagenomic sequencing of the seagrass, Zostera marina, include a novel chytrid in the order Lobulomycetales

**DOI:** 10.1099/mgen.0.001845

**Published:** 2026-09-23

**Authors:** Cassandra L. Ettinger, Jonathan A. Eisen, Jason E. Stajich

**Affiliations:** 1 Department of Microbiology and Plant Pathology, University of California, Riverside, CA, USA; 2 Genome Center, University of California, Davis, CA, USA; 3 Department of Evolution and Ecology, University of California, Davis, CA, USA; 4 Department of Medical Microbiology and Immunology, University of California, Davis, Davis, CA, USA; 5 Institute for Integrative Genome Biology, University of California, Riverside, Riverside, CA, USA

**Keywords:** Bacillariophyta, Chytridiomycota, eelgrass, genome assembly, genome annotation, Lobulomycetales, marine fungi, metagenomics, microbial eukaryotes, Prymnesiophyceae, *Zostera marina*

## Abstract

Fungi play pivotal roles in terrestrial ecosystems as decomposers, pathogens and endophytes, yet their significance in marine environments is often understudied. Seagrasses, as globally distributed marine flowering plants, have critical ecological functions but knowledge about their associated fungal communities remains relatively limited. Previous amplicon surveys of the fungal community associated with the seagrass, *Zostera marina,* have revealed an abundance of potentially novel chytrids. In this study, we employed deep metagenomic sequencing to extract metagenome-assembled genomes (MAGs) from these chytrids and other microbial eukaryotes associated with *Z. marina* leaves. Our efforts resulted in the recovery of five eukaryotic MAGs, including a single fungal MAG in the order Lobulomycetales (65% BUSCO completeness), three MAGs representing diatoms in the family Bacillariaceae (93%, 70% and 31% BUSCO completeness) and a single MAG representing a haptophyte alga in the genus *Prymnesium* (40% BUSCO completeness). Whole-genome phylogenomic assessment of these MAGs suggests they all largely represent undersequenced and possibly novel eukaryotic lineages. Of particular interest, the chytrid MAG was placed within the order Lobulomycetales, consistent with the identity of the dominant chytrid from previous *Z. marina* amplicon survey results. Annotation of this MAG yielded 5,650 gene models, of which 77% shared homology with current databases. Within these gene models, we predicted 121 carbohydrate-active enzymes (CAZymes) and 393 secreted proteins (103 cytoplasmic effectors, 30 apoplastic effectors). Exploration of orthologs between the Lobulomycetales MAG and existing Chytridiomycota genomes has revealed a landscape of high-copy gene families related to host recognition and interaction. Further machine learning analyses based on CAZyme composition classified this MAG’s CAZyme profile as most consistent with a symbiotic lifestyle. Overall, these five eukaryotic MAGs represent substantial genomic novelty and valuable community resources, contributing to a deeper understanding of the roles of fungi and other microbial eukaryotes in the larger seagrass ecosystem.

**Impact Statement**

Microbial eukaryotes, in particular fungi, have critical roles associated with land plants, but their association with marine plants, like seagrasses, remains largely uncharacterized. This study generates metagenome-assembled genomes (MAGs) of microbial eukaryotes from seagrass leaf tissue, recovering five draft genomes representing diatoms, algae and a chytrid fungus in the poorly understood order Lobulomycetales. With only two prior genomes available for this entire fungal order, this chytrid MAG represents a substantial advance in genomic coverage of an ecologically important but deeply understudied lineage. Functional annotation and comparative genomics of this MAG reveal a gene repertoire consistent with a symbiotic lifestyle, shifting our understanding of the possible ecological roles of chytrid fungi in seagrass ecosystems. Together, these five MAGs provide foundational genomic resources for a community of researchers working to understand the role of microbial eukaryotes in seagrass health, carbon cycling and ecosystem resilience in the face of global change.

## Data Summary

 The raw metagenomic sequencing data were deposited at GenBank under accession no. PRJNA1140276. MAGs and annotations under this BioProject at DDBJ/ENA/GenBank under accession no. JBKBCY000000000-JBKBDC000000000. The 18S rRNA gene obtained for SGEUK-03 was deposited at GenBank under accession no. PQ146498. All code used in this work has been deposited on Github (casett/ZM_Euk_MAGs) and archived in Zenodo (DOI: 10.5281/zenodo.14278507).

## Introduction

Fungi are key contributors in terrestrial ecosystems, functioning as decomposers, pathogens and endophytes, but their importance in marine environments remains underexplored [1–3]. While over 120,000 species of terrestrial fungi have been documented, only ~2,100 marine fungal species have been formally described, although the actual diversity is believed to be much higher [4–7]. Recent research has shed light on the distribution, activity and biomass of marine fungi, with current estimates suggesting that fungi contribute 0.19–0.21 Gt C to the biomass of the open ocean [8–14]. However, host-associated fungal communities in marine ecosystems, including those associated with marine plants such as seagrasses, are still largely understudied.

Seagrasses are fully submerged flowering plants and vital foundation species in coastal ecosystems globally. *Zostera marina*, commonly known as eelgrass, is a widely distributed species that plays a crucial role in providing essential ecosystem services across coastal regions in the Northern Hemisphere [15–17]. Seagrasses like *Z. marina* are home to a diverse range of microbial eukaryotes, including diatoms, dinoflagellates, algae, oomycetes, protists and fungi [18–24]. Studies have shown that fungi represent only a small fraction of the total epiphytic eukaryotic community associated with *Z. marina*, with diatoms, oomycetes and dinoflagellates among the dominant groups [25–27].

Although culture-based studies have explored fungal diversity associated with *Z. marina*, these approaches are limited by the challenges of growing marine fungi in laboratory settings [28–32]. Advances in DNA-based, culture-independent sequencing techniques have allowed for a more comprehensive characterization of seagrass-associated fungal communities, including their community assembly and biogeographical patterns [25, 33–39]. Despite this progress, many seagrass-associated fungi, particularly those from Chytridiomycota lineages, remain difficult to taxonomically classify due to biases in reference databases, which are heavily skewed towards terrestrial fungi [25, 36, 38, 39]. This is unsurprising as a limited number of lineages of marine Chytridiomycota have been described through culture-based methods, and few have sequenced genomes [6, 7, 40, 41]. Nevertheless, marine Chytridiomycota are consistently recovered in DNA-based surveys and are often dominant members of seagrass-associated fungal communities [14, 25, 34, 36, 42].

Chytridiomycota in the order Lobulomycetales have previously been observed in high abundance on and inside *Z. marina* leaves [36], and members of this group are part of the *Z. marina* core mycobiome [25]. The Lobulomycetales are a relatively understudied group of chytrid fungi that have been found to span terrestrial, freshwater and marine ecosystems as both parasites and saprobes [43]. The two sequenced genomes from this group represent *Lobulomyces angularis,* which was isolated from an acidic freshwater lake [44], and *Clydaea vesicula,* which was isolated from soil underneath Eucalyptus trees [43]. During the initial description of the Lobulomycetales, the authors noted that due to the difficulty in isolating members of this group, even for ‘chytrid specialists’, that they expected most members would need to be identified and described from molecular data [43].

While previous work identified high relative abundance of chytrid fungi associated with seagrasses, particularly members of the Lobulomycetales, we still have little understanding of the functional role of these enigmatic aquatic chytrids [25, 36, 45]. To address this, we used a metagenomic sequencing approach on DNA that was previously extracted from *Z. marina* leaves in Ettinger and Eisen [36] and was reported to have a high relative abundance of Lobulomycetales chytrid fungi. We then used this metagenomic sequencing data to: (i) generate a set of high-quality draft eukaryotic metagenome-assembled genomes (MAGs), (ii) phylogenetically place in the tree of life and functionally annotate all high-quality draft eukaryotic MAGs generated and (iii) use comparative genomics approaches to explore hypotheses of the possible function and ecology of any fungal MAGs.

## Methods

### Sequence generation

DNA was extracted from epiphytic washes from healthy green *Z. marina* leaves as part of previous work focused on characterizing the mycobiome using high-throughput sequencing of the ITS2 region [36]. Three DNA extracts from that work were selected based on predicted relative abundances of an unclassified chytrid in the order Lobulomycetales (identified as amplicon sequence variant SV8), with two samples (108A and 109A) having relatively high abundance of this putative taxon and one sample having a low abundance of this taxon (140A). These samples with variable abundance of SV8 were chosen to aid binning algorithms, which can utilize differential coverage to aid in metagenomic bin recovery [46, 47]. Further, the prior work sampled both epiphytic washes and endophytic leaf fractions and recovered the target Lobulomycetales chytrid in both [36]; the DNA from epiphytic washes was chosen here to reduce the relative burden of host *Z. marina* DNA in the resulting metagenomic libraries. DNA was provided to the UC Davis Genome Center DNA Technologies Core for library preparation and sequencing. DNA libraries were sequenced on an Illumina HiSeq4000 to generate 150 bp paired-end reads.

### Sequence processing

Sequence reads were trimmed using bbduk v. 37.68 [48] with the following parameters: qtrim=rl trimq=10 maq=10. We then mapped against and removed any reads from the metagenomes matching the available genome for *Z. marina* v. 3.1 [49] using bowtie2 v. 2.4.5 [50] and samtools v. 1.11 [51]. The remaining reads from all three metagenomic samples were co-assembled using MEGAHIT v. 1.2.9 [52]. We ran Whokaryote v. 1.1.2 [53], which uses a random forest classifier to predict the eukaryotic origin of contigs. Whokaryote was run with the parameter --minsize 1 kbp and we chose to include predictions from Tiara v. 1.0.3 [53, 54], which uses deep learning to further identify eukaryotic contigs.

The anvi’o v. 7.2 workflow [55] was used to identify and assess eukaryotic MAGs. This workflow was executed twice: once for the entire co-assembly and once for the subset of the co-assembly containing only the Whokaryote predicted eukaryotic contigs. Briefly, this workflow involved obtaining read coverage for each metagenomic sample against the co-assembly using bowtie2 v. 2.4.5 [50] and samtools v. 1.11 [51]. Next ‘anvi-gen-contigs-database’ was used to generate a contig database from the co-assembly and gene calls in the co-assembly were predicted using Prodigal v. 2.6.3 [56]. Single-copy bacterial [57], archaeal [57] and protista [58] genes were then predicted using HMMER v. 3.2.1 [59], and ribosomal RNA genes were predicted using barrnap [60]. Each gene call was then assigned a putative taxonomy using Kaiju v. 1.8.2 [61] with the NCBI blast non-redundant protein database (nr), including fungi and microbial eukaryotes v. 2020-05-25. For each metagenomic sample, an individual anvi’o profile was constructed using ‘anvi-profile’ for contigs >1 kbp with the ‘–cluster-contigs’ option. Several automatic binning algorithms were then employed to generate a preliminary set of MAGs including MetaBAT2 v. 2.15, MaxBin v. 2.2.1, BinSanity v.0.5.4 and CONCOCT v. 1.1.0 [62–65]. All predicted MAG bins were then assessed with eukCC v. 2.0 [66] for eukaryotic completeness and redundancy.

To obtain a final representative set of eukaryotic MAGs, dereplication was performed using dRep v. 3.2.2 [67] by providing the eukCC completeness estimates. This step involved combining bins identified from both anvi’o workflow runs (the results from the entire co-assembly and the subset of eukaryotic contigs). The minimum completeness threshold for dRep was set to 1 to ensure a comprehensive representation of eukaryotic MAGs, which often have lower completeness compared to bacterial MAGs.

Representative MAGs with >30% eukaryotic completeness were assessed using anvi-refine. A completeness threshold of >30% has been previously used as a cut-off for high-quality eukaryotic draft MAGs [68]. Further assessment and decontamination of these eukaryotic MAGs was performed using the BlobTools2 v. 4.1.4 [69] workflow. This workflow involves assigning taxonomy based on searches against the UniProt Reference Proteomes database (v. 2021_04) to each contig using diamond (v. 2.1.0) and against the nt database (downloaded 2023-03-21) using command-line blast v. 2.2.30+ [70, 71]. Next, coverage of each individual metagenomic sample against each eukaryotic bin was calculated with Minimap2 v. 2.24 [72] and sorted using samtools v. 1.11 [51]. Finally, the BlobToolKit Viewer was used to visualize the G+C content, read coverage and predicted taxonomies of contigs to identify contaminants. To be conservative, any contigs that were taxonomically identified as viral, bacterial or archaeal were removed from the eukaryotic bins.

After removing potential contamination, we ran EUKulele v. 1.0.2 [73] to assess and refine the initial taxonomic assignments for each bin. We also used BUSCO v. 5.3.2 [74] to assess completion of the de-contaminated bins in ‘genome’ mode using the eukaryotic_odb10 set, and as appropriate the fungi_odb10 or stramenopiles_odb10 sets.

### Annotation

All high-quality (>30% complete) draft eukaryotic MAGs that were obtained were annotated. Draft MAG assemblies were screened for contaminant vectors using the ‘vecscreen’ option in the Automatic Assembly of the Fungi pipeline v. 0.4.1 [75]. Repetitive regions were *de novo* identified and soft masked prior to MAG annotation using RepeatModeler v. 2.0.1 [76] and RepeatMasker v. 4-1-1 [77].

Draft eukaryotic MAGs were annotated using the Funannotate pipeline v. 1.8.14 [78]. Funannotate incorporates multiple software algorithms for eukaryotic gene prediction, including Augustus v. 3.4.0, GeneMark-ETS v. 4.71, GlimmerHMM v. 3.0.4 and SNAP v. 2013_11_29 [79–83], and then generates consensus gene models using EVidenceModeler v. 1.1.1 [84]. For two MAGs (SGEUK-04 and SGEUK-05) where GeneMark-ETS was unable to run due to assembly fragmentation, we used –weights genemark:0 during prediction to skip use of this software. Funannotate also uses tRNAscan v. 2.0.11 to predict tRNAs [85]. Annotation of consensus gene models by Funannotate is based on searches against Pfam v. 35.0 [86] and dbCAN v. 9.0 [87, 88] using HMMER v.3 [59] and against MEROPS v. 12.0 [89], eggNOG v. 2.1.9 [90], InterProScan v. 5.51–85.0 [91] and UniProt v. 2022_05 [92] using diamond v. 2.0.15 [93]. Transmembrane proteins are further predicted using Phobius v. 1.01 [94] and secreted proteins using SignalP v. 5.0b [95]. For the fungal MAG (SGEUK-03), AntiSMASH v. 6.1.1 was run to predict biosynthetic gene clusters [96], and EffectorP v. 3.0 was run on putative secreted proteins to predict apoplastic and cytoplasmic effectors [97]. We also used CATAStrophy v. 0.1.0 [98], a classification method based on carbohydrate-active enzyme (CAZyme) patterns from filamentous fungal plant pathogens, to predict the possible trophic strategy of SGEUK-03. CATAStrophy was run in a Google Collab implementation using dbCAN v. 10 [87, 88].

BUSCO v. 5.3.2 [74] was then used to assess completion of MAG annotations in ‘protein’ mode using the eukaryotic_odb10 set, and as appropriate the fungi_odb10 or stramenopiles_odb10 sets.

### Phylogenetic assessment

To examine the phylogenetic placement of all five high-quality draft eukaryotic MAGs generated, we carried out analysis of three groups: stramenopiles (SGEUK-01, SGEUK-02, SGEUK-04), fungi (SGEUK-03) and haptista (SGEUK-05). For each group, we obtained gene models from annotated genomes that were publicly available in NCBI GenBank (Tables S1–S3, available in the online Supplementary Material). Given the limited availability of annotated genomes for haptista (four genomes at the time of this work), we also incorporated publicly available transcriptomic data from METdb [99–101].

For stramenopiles, the final dataset for phylogenomic analyses included SGEUK-01, SGEUK-02, SGEUK-04 and 153 annotated genomes representing the following groups: Oomycota (*n*=120), Bacillariophyta (*n*=11), Bigyra (*n*=8), Pelagophyceae (*n*=7), Eustigmatophyceae (*n*=3), Phaeophyceae (*n*=2), Synurophyceae (*n*=1) and Xanthophyceae (*n*=1) (Table S1) [102–164]. For fungi, the final dataset for comparative genomics and phylogenomic placement included SGEUK-03 and 98 annotated genomes from the following phyla: Chytridiomycota (*n*=71), Blastocladiomycota (*n*=10), Neocallimastigomycota (*n*=8), Ascomycota (*n*=4), Monoblepharidomycota (*n*=3), Olpidiomycota (*n*=1) and Basidiomycota (*n*=1) (Table S2) [165–186]. For haptista, the final dataset for phylogenomic placement included SGEUK-05, 47 annotated genomes and transcriptomes from members of the orders Phaeocystales (*n*=14), Isochrysidales (*n*=14), Prymnesiales (*n*=9), Pavlovales (*n*=7), Coccolithales (*n*=2), Coccosphaerales (*n*=1) and Haptophyta *incertae sedis* (*n*=1) (Table S3) [101, 162, 187–189].

To place MAGs in a phylogenetic context, the PHYling_unified (https://github.com/stajichlab/PHYling_unified) pipeline was used to generate a protein alignment of each MAG to close relatives based on preliminary taxonomic assignment (i.e. the datasets described in Tables S1-S3). This pipeline utilizes HMMER v.3 [59] and ClipKIT [190] to search for, build and trim a concatenated alignment based on gene set. Here, we used the most relative BUSCO gene set for each dataset, e.g. the BUSCO fungi_odb10 for SGEUK-3, eukaryotic_odb10 for SGEUK-05 and stramenopiles_odb10 for SGEUK-01, SGEUK-02 and SGEUK-04. Maximum-likelihood phylogenies were built from these concatenated alignments using IQ-TREE2 v.2.2.6 [191], with the -p option to indicate gene partitions [192] and the -m MFP option to run ModelFinder Plus to identify and use the optimal evolutionary model for each partition [193]. The resulting phylogenetic trees were imported into R v. 4.3.0 [194] and visualized using ggtree v. 3.8.2 [195].

Given that 18S rRNA sequences often do not end up in MAG bins [196] and to further refine taxonomy for the MAGs, we used phyloflash v. 3.4 [197] to assemble and identify the 18S rRNA gene sequences in the metagenomes. While we were able to identify an 18S rRNA gene for the fungal MAG (SGEUK-03), we were not able to clearly and unambiguously link 18S rRNA genes to the stramenopile or haptophyte MAGs. We then identified closely related sequences to the 18S rRNA gene sequence for the fungal MAG using NCBI’s Standard Nucleotide blast with default settings [71]. These results were then used to guide a literature search to identify additional sequences for inclusion during phylogenetic reconstruction (Table S4) [43, 198–209]. These 18S rRNA gene sequences were then aligned using SSU-ALIGN v. 0.1.1 [210] and trimmed using trimAl v. 1.4 with the -gappyout method [211]. A maximum-likelihood phylogeny was built from this alignment using IQ-TREE2 v.2.2.6 [191], with the -p option to indicate gene partitions [192] and the -m MFP option to run ModelFinder Plus to identify and use the optimal evolutionary model for each partition [193]. The resulting phylogenetic tree was imported into R and visualized using ggtree v. 3.8.2 [195].

### Comparative genomics of chytrid MAG

OrthoFinder v. 2.5.4 [212] was used to predict phylogenetic hierarchical orthogroups (HOGs), or gene families, across Chytridiomycota and related lineages (Table S1). Given the little genomic information available for close relatives of SGEUK-03, we focused our analyses on HOGs at the root node (i.e. all HOGs). Due to the incomplete nature of MAGs, we chose to focus comparative analyses on high-copy HOGs and did not explore gene loss. High-copy HOGs (≥5 copies in SGEUK-03) were visualized in R using the pheatmap v. 1.0.12 package [213].

## Results

### Successful recovery and preliminary annotation of five eukaryotic MAGs

In total, we recovered five eukaryotic MAGs that were high-quality (>30% complete) and proceeded with taxonomic identification and annotation for all five. Briefly, we note that bins resulting from the co-assembly containing only Whokaryote identified eukaryotic contigs had lower contamination, although at the expense of overall completion, compared to their entire co-assembly counterparts. Among the five dereplicated MAGs, only one MAG (SGEUK-05) was not obtained from the eukaryotic co-assembly.

To perform preliminary taxonomic assignment of MAGs, we used EUKulele which reports the relative proportion of all the proteins within an MAG that have annotations that agree at a particular taxonomic level. Using a conservative cut-off of >70% proportional agreement, EUKulele predicted that we had three Bacillariophyta MAGs (SGEUK-01 : 89.60%, SGEUK-02 : 78.39% and SGEUK-04 : 75.08%), one Eukaryota MAG (SGEUK-03 : 94.24%) and one Prymnesiophyceae MAG (SGEUK-05 : 82.24%).

These eukaryotic draft MAGs were fairly fragmented (i.e. with total contigs ranging from 1,074 to 11,398) likely due to their relatively low mean coverage (i.e. ranging from 3.47 to 7.71×). Briefly, we discuss the general characteristics of the assembly and annotation of each draft MAG (Table 1):

- SG-EUK01 (Bacillariophyta) spans 32.54 Mbp with 3.46× coverage, distributed across 2,172 contigs with an N50 of 20,103 bp. Initial EukCC completion and contamination estimates were 84.5 and 5.7%, respectively. After removing 21 contigs flagged as bacterial contamination by BlobTools, BUSCO analysis using the stramenopiles_odb10 dataset indicated a high completeness of 93.0%. Annotation with Funannotate resulted in 13,603 gene models, including 13,520 mRNA genes and 83 tRNA genes, with 75.92% of gene models having EggNog database hits. BUSCO protein estimates were consistent with the genome completeness, showing 91.0% of the expected single-copy orthologs present using the stramenopiles_odb10 set.
- SG-EUK02 (Bacillariophyta) is 28.49 Mbp in length with 5.37× coverage, spanning 5,479 contigs and an N50 of 5,715 bp. EukCC estimated 64.39% completeness and 13.96% contamination, lower in completeness but higher in contamination than SG-EUK01. We subsequently removed 92 contigs identified as bacterial contamination using BlobTools. BUSCO estimates show 70.0% completeness using the stramenopiles_odb10 dataset. Annotation resulted in 10,889 gene models, including 10,868 mRNA genes and 21 tRNA genes, with 76.43% of gene models having EggNog database hits. BUSCO estimates are aligned with genome completeness, showing 69.0% of single-copy orthologs present using the stramenopiles_odb10 set.
- SG-EUK03 (Eukaryota) is a smaller MAG at 12.11 Mbp with 7.71×x coverage, distributed across 1,074 contigs with an N50 of 15,102 bp. EukCC predicted completion and contamination at 56.96% and 1.74%, respectively. After removing 10 bacterial contigs identified by BlobTools, BUSCO analysis revealed moderate completeness, with 65.1% using the eukaryota_odb10 dataset and 51.6% using the fungi_odb10 dataset. Annotation resulted in 5,650 gene models, including 5,630 mRNA genes and 20 tRNA genes, with 76.58% of gene models having EggNog database annotation hits. Notably, 393 genes (6.96%) were predicted to be secreted, including 133 potential effectors (103 cytoplasmic, 30 apoplastic), and 121 genes (2.14%) had CAZyme domains, including 65 glycosyltransferases (GTs), 26 glycoside hydrolases (GHs), 13 carbohydrate esterases (CEs), 8 polysaccharide lyases (PLs), 7 auxiliary activities and 2 carbohydrate-binding modules (CBMs) (Fig. S1, Table S5). CATAStrophy classified SGEUK-03’s CAZyme content as consistent with a symbiotic lifestyle. BUSCO protein estimates indicated 65.9% completeness with eukaryota_odb10 and 57.0% with fungi_odb10, outperforming the genome completeness estimates.
- SG-EUK04 (Bacillariophyta) spans 19.6 Mbp with 4.04× coverage, spread over 11,398 contigs with an N50 of 1,790 bp. EukCC estimated a relatively low completeness of 35.9% and contamination of 5.98%, lower in completeness compared to SG-EUK01 and SG-EUK02. We removed 197 contigs identified as potential bacterial contaminants. BUSCO estimates using the stramenopiles_odb10 dataset indicated that only 31.0% of single-copy orthologs were present and complete. Annotation resulted in 8,359 gene models, including 8,343 mRNA genes and 16 tRNA genes, with 64.77% of gene models having EggNog database hits. BUSCO protein estimates are aligned with the genome completeness, with 30.0% of expected single-copy orthologs present using the stramenopiles_odb10 set.
- SG-EUK05 (Prymnesiophyceae) is the largest of the MAGs, spanning 59.4 Mbp with 5.12× coverage, distributed across 9,735 contigs with an N50 of 6,756 bp. Initial EukCC estimates placed completion at 34.0% and contamination at 3.4%. Following the removal of 197 bacterial contigs, BUSCO estimates showed that 40.0% of expected orthologs were present using the eukaryota_odb10 dataset. Annotation resulted in 15,098 gene models, including 15,075 mRNA genes and 23 tRNA genes, with 56.78% of gene models having EggNog database annotation hits. Repeats represented 18.63% of the genome length, much higher than for any of the other draft MAGs. BUSCO protein estimates performed slightly worse than the genome completeness, with only 34.5% of the expected single-copy orthologs present and complete.

**Table 1.** Eukaryotic MAG assembly and annotation statistics

Bin ID	Phylum	Putative taxonomy	EukCC completion	Genome size (Mbp)	No. of contig	N50 (bp)	G+C content (mol%)	Mean coverage (×)	Repetitive region (%)	No. of gene model	No. of mRNA	No. of tRNA	Genes with InterProScan hit (%)	Genes with eggNOG hit (%)	Genes with GO term (%)	Genes with CAZyme hit (#)	NCBI accession
SGEUK-01	Gyrista	Bacillariaceae sp.	84.05	32.54	2,172	20,103	45.84	3.46	2.9	13,603	13,520	83	66.54	75.92	44.34	153	JBKBDC000000000
SGEUK-02	Gyrista	Bacillariaceae sp.	64.39	28.49	5,479	5,715	48.42	5.37	1.44	10,889	10,868	21	65.97	76.43	45.97	132	JBKBDB000000000
SGEUK-03	Chytridiomycota	Lobulomycetales sp.	56.96	12.11	1,074	15,102	48.57	7.71	1.7	5,650	5,630	20	77.38	76.58	58.48	121	JBKBDA000000000
SGEUK-04	Gyrista	Bacillariaceae sp.	35.9	19.6	11,398	1,790	48.43	4.04	1.55	8,359	8,343	16	53.08	64.77	34.08	44	JBKBCZ000000000
SGEUK-05	Haptista	*Prymnesium* sp.	34	59.4	9,735	6,756	53.36	5.12	18.63	15,098	15,075	23	54.65	56.78	36.01	175	JBKBCY000000000

We identified five high-quality (> 30% complete) eukaryotic MAGs associated with *Z. marina* leaves. Here, we list their proposed taxonomy, report on various genome and annotation quality statistics and provide the NCBI accession information for each MAG.

Overall, despite fragmentation, these five eukaryotic MAGs exhibited varying levels of genome and annotation completeness. The Bacillariophyta MAGs, particularly SG-EUK01 and SG-EUK02, displayed higher genome completeness, reflecting robust reconstructions of their genomic landscapes. SG-EUK03 showed a more moderate genome and annotation completeness. SG-EUK04 and SG-EUK05 are more fragmented, although still represent valuable insight into the genomes and functions of these microbial eukaryotes.

### Phylogenetic placement of eukaryotic MAGs puts them in the diatom, fungal and haptophyte trees of life

Following MAG recovery and functional annotation, we constructed phylogenetic trees to refine the taxonomic placement of all five draft MAGs. Using BUSCO protein sets and guided by preliminary taxonomic assignments from EUKulele, we built three trees: one for Bacillariophyta, one for fungi and one for Haptista.

SGEUK-01, SGEUK-02 and SGEUK-04 were placed into the Bacillariophyta tree, and clustered among diatom lineages in the Bacillariaceae family, with SG-EUK02 and SG-EUK04 forming a clade that is sister to *Nitzschia inconspicua*, while SG-EUK01 formed a novel branch serving as outgroup to the clade representing all members of the Bacillariaceae family (Fig. 1). While SGEUK-01 had very similar BUSCO completion estimates compared to annotated close relatives, both SGEUK-02 and SGEUK-04 likely paint a more incomplete picture of the functional landscape of these organisms. All three MAGs have genomes and annotations on the smaller side with publicly available genomes for members of the Bacillariaceae family ranging from 27.23 to 99.71 Mbp in size [123, 214] and available annotations ranging in gene count from 11,972 to 39,085 genes [123, 139].

**Fig. 1.**
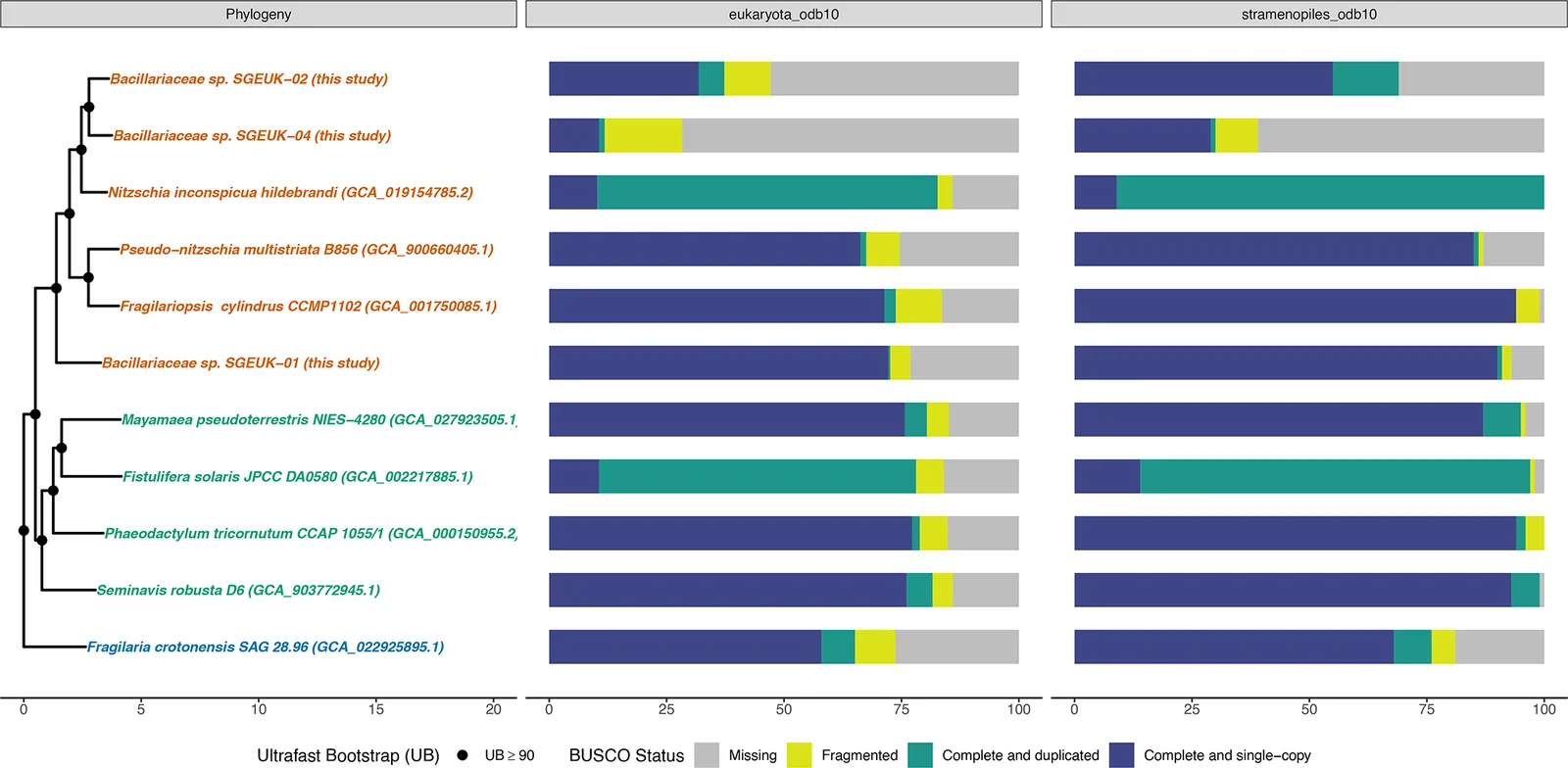
SGEUK-01, SGEUK-02 and SG-EUK04 are phylogenetically placed in the family Bacillariaceae. From left to right, first, a maximum-likelihood phylogeny that shows the placement of SGEUK-01, SGEUK-02 and SG-EUK04 in the gyrista tree of life among diatom lineages. This tree was generated using IQ-TREE2 on an alignment of BUSCO stramenopiles_odb10 HMMs constructed using the PHYling_unified pipeline. Taxon labels in the phylogeny are shown coloured by their assigned taxonomic family (orange, Bacillariaceae; green, Naviculaceae; blue, Fragilariaceae). Next, in association with this phylogeny, bar charts of the BUSCO ‘protein’ completion status for the eukaryota_odb10 and stramenopiles_odb10 sets is shown. Bars show the percentage of genes found in each genome annotation as a percentage of the total gene set and are coloured by BUSCO status (missing, grey; fragmented, yellow; complete and duplicated, green; complete and single copy, blue).

SGEUK-03 was placed into the fungal tree, falling amongst Chytridiomycota lineages (Fig. 2). The MAG was placed sister to a clade containing *L. angularis* and *C. vesicula*, which both belong to the order Lobulomycetales, an undersequenced group of chytrids. *Quaeritorhiza haematococci*, a parasitic chytrid of algae [215], serves as an outgroup to the Lobulomycetales clade containing these three taxa. It falls in the same taxonomic class as Lobulomycetales, the Chytridiomycetes, but its phylogenetic placement in an order is uncertain. Regardless, it has been shown to be sister to the Lobulomycetales in other phylogenetic studies [216]. The two available Lobulomycetales genomes are similarly fragmented to SGEUK-03, with *L. angularis* represented by 1,253 scaffolds with an N50 of 26,617 bp and *C. vesicula* spanning 2,291 scaffolds with an N50 of 14,665 bp [172]. However, both their genomes (24.65–24.94 Mbp) and annotations (8,737–9,208 genes) are larger than SGEUK-03 which is in line with its lower BUSCO completion scores (Fig. 2).

**Fig. 2.**
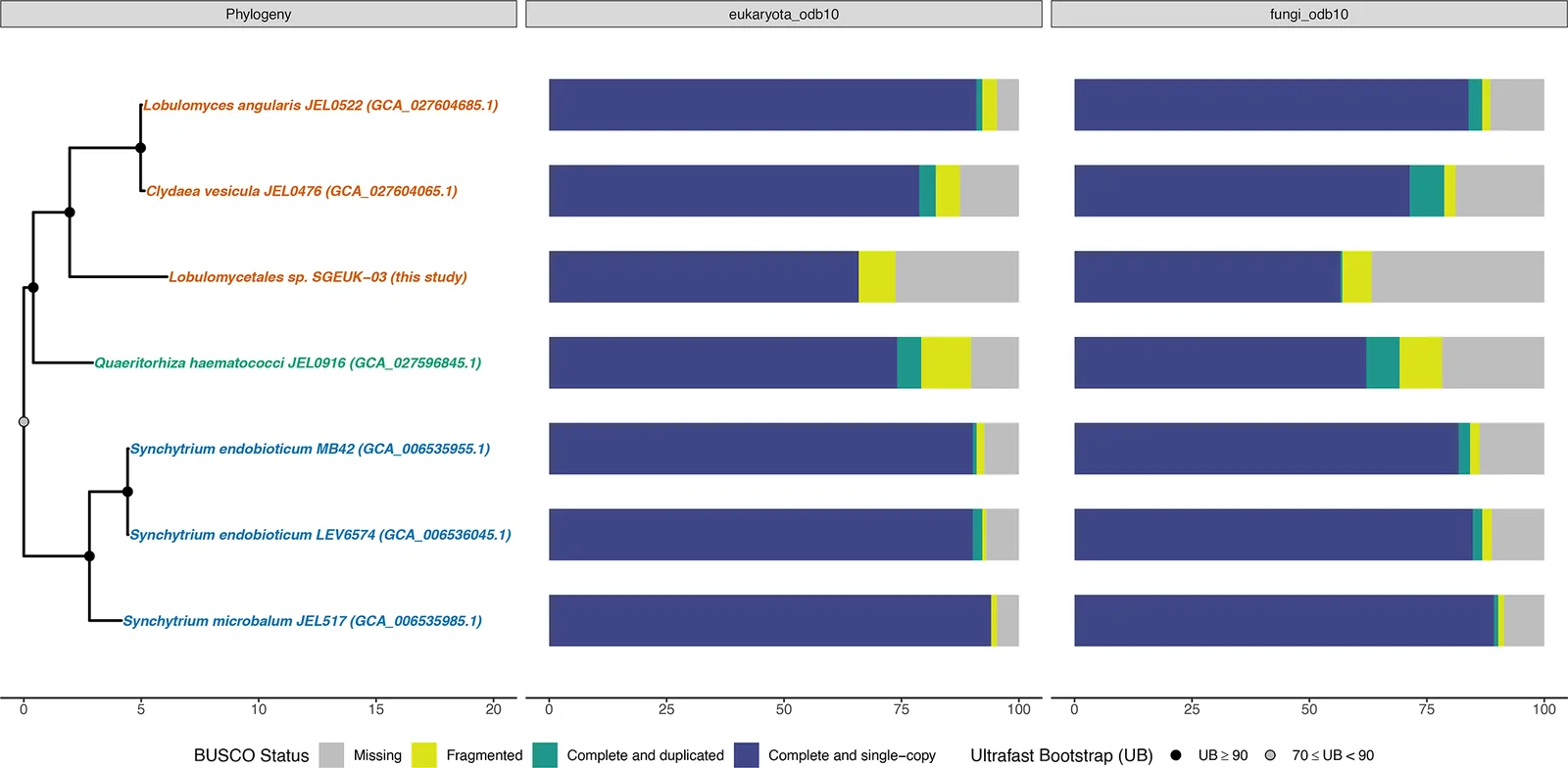
SGEUK-03 is phylogenetically placed in the order Lobulomycetales. From left to right, first, a maximum likelihood phylogeny that shows the placement of SGEUK-03 in the fungal tree of life. This tree was generated using IQ-TREE2 on an alignment of BUSCO fungi_odb10 HMMs constructed using the PHYling_unified pipeline. Taxon labels in the phylogeny are shown coloured by their assigned taxonomic order (orange = Lobulomycetales, green = Chytridiomycetes *incertae sedis*, blue = Synchytriales). Next, in association with this phylogeny, bar charts showing the BUSCO ‘protein’ completion status for the eukaryota_odb10 and fungi_odb10 sets are shown. Bars show the percentage of genes found in each genome annotation as a percentage of the total gene set and are coloured by BUSCO status (missing, grey; fragmented, yellow; complete and duplicated, green; complete and single copy, blue).

To further confirm and improve placement of SGEUK-03 among the Lobulomycetales, we used phyloFlash to recover an 18S rRNA gene from the metagenomic dataset likely belonging to this MAG and then placed it in the context of available 18S rRNA genes representing this chytrid lineage. This 18S rRNA gene phylogeny confirmed placement of SGEUK-03 among the Lobulomycetales, and further placed it within the novel SW-I clade, an aquatic clade of Lobulomycetales largely described from DNA survey datasets (Fig. 3). SGEUK-03 is sister to a sequence from deep sea marine sediment [199] and is in a clade with other sequences from marine surveys [198]. This taxonomic placement matches that previously obtained for the dominant amplicon sequence variant (SV8) from ITS2 amplicon profiling of the same samples, supporting that SGEUK-03 represents the chytrid fungus previously identified to be abundant on *Z. marina* leaves [36].

**Fig. 3.**
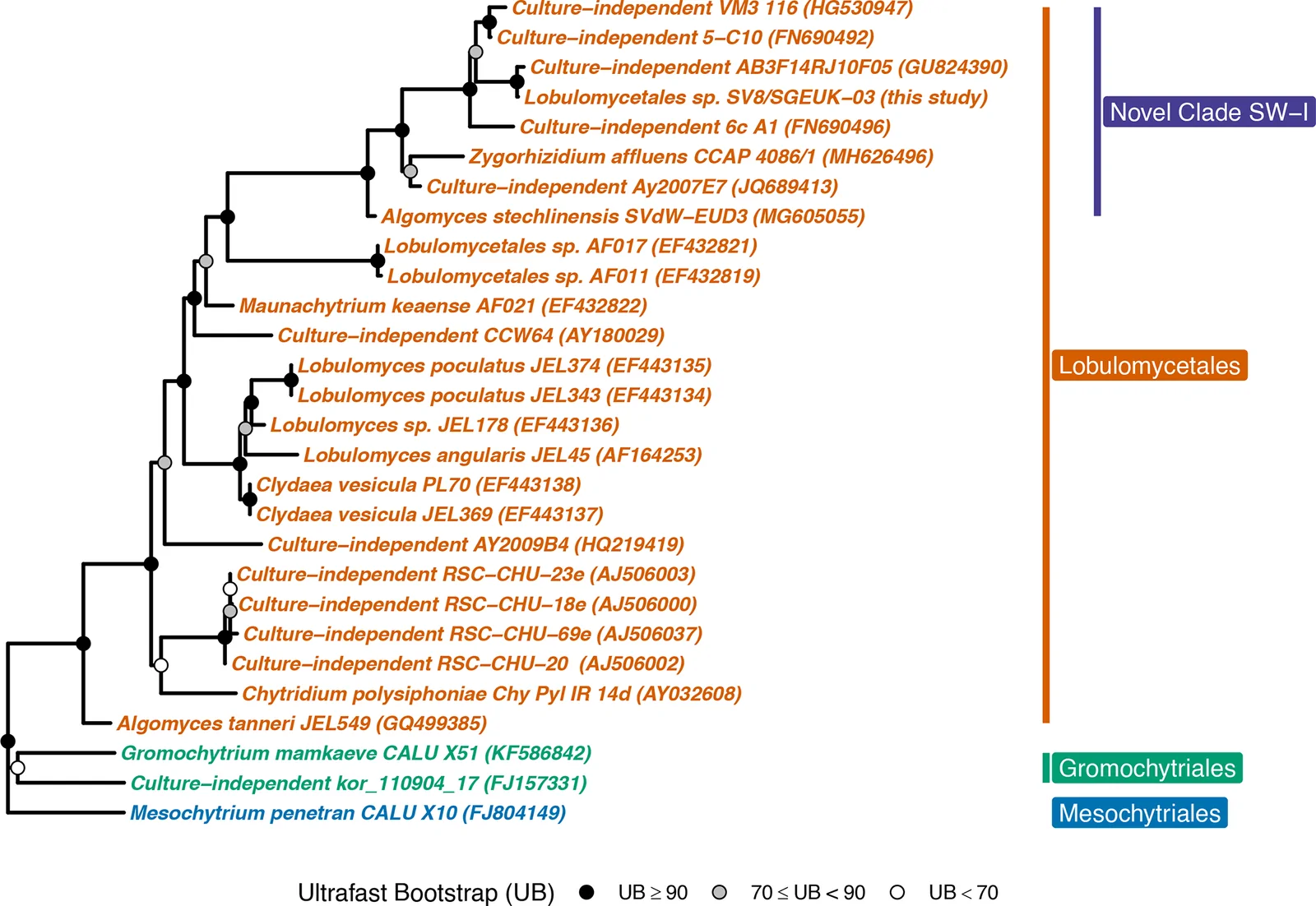
SGEUK-03 18S ribosomal RNA gene tree confirms placement in the Lobulomycetales in Novel Clade SW-I. Given the lack of closely related genomes to SGEUK-03, we used phyloFlash to assemble the 18S rRNA gene associated with SGEUK-03 and then aligned it to close relatives using SSU-align. The alignment was trimmed with trimAl, and IQ-TREE2 was run with ModelFinderPlus, to identify and use the optimal evolutionary model, and 1,000 ultrafast bootstraps. Taxon labels in the phylogeny are coloured and indicated by bars based on their assigned taxonomic order. Additionally, Novel Clade SW-I is also indicated by a bar. Displayed at each node as a circle in the tree are the ultrafast bootstrap values (e.g. a black circle represents bootstrap values greater or equal to 90%, a grey circle represents bootstrap values greater or equal to 70%, a white circle represents bootstrap values less than 70%).

SGEUK-05 was placed into the haptophyta tree, among the Prymnesiales order and sister to the transcriptome of the phytoplankton, *Prymnesium parvum* (Fig. 4). SGEUK-05 and *P. parvum* were sister to a clade of transcriptomes from *Chrysochromulina polylepis* which are also members of the Prymnesiaceae family. *Chrysochromulina* genomes are 59.07–65.75 Mbp [189], although other members of the Prymnesiales have genomes up to ~167.68 Mbp [187]. Given the relatively low completion of SGEUK-05, despite its larger size of 59.4 Mbp, it is likely we are only capturing less than half of its genomic and functional space.

**Fig. 4.**
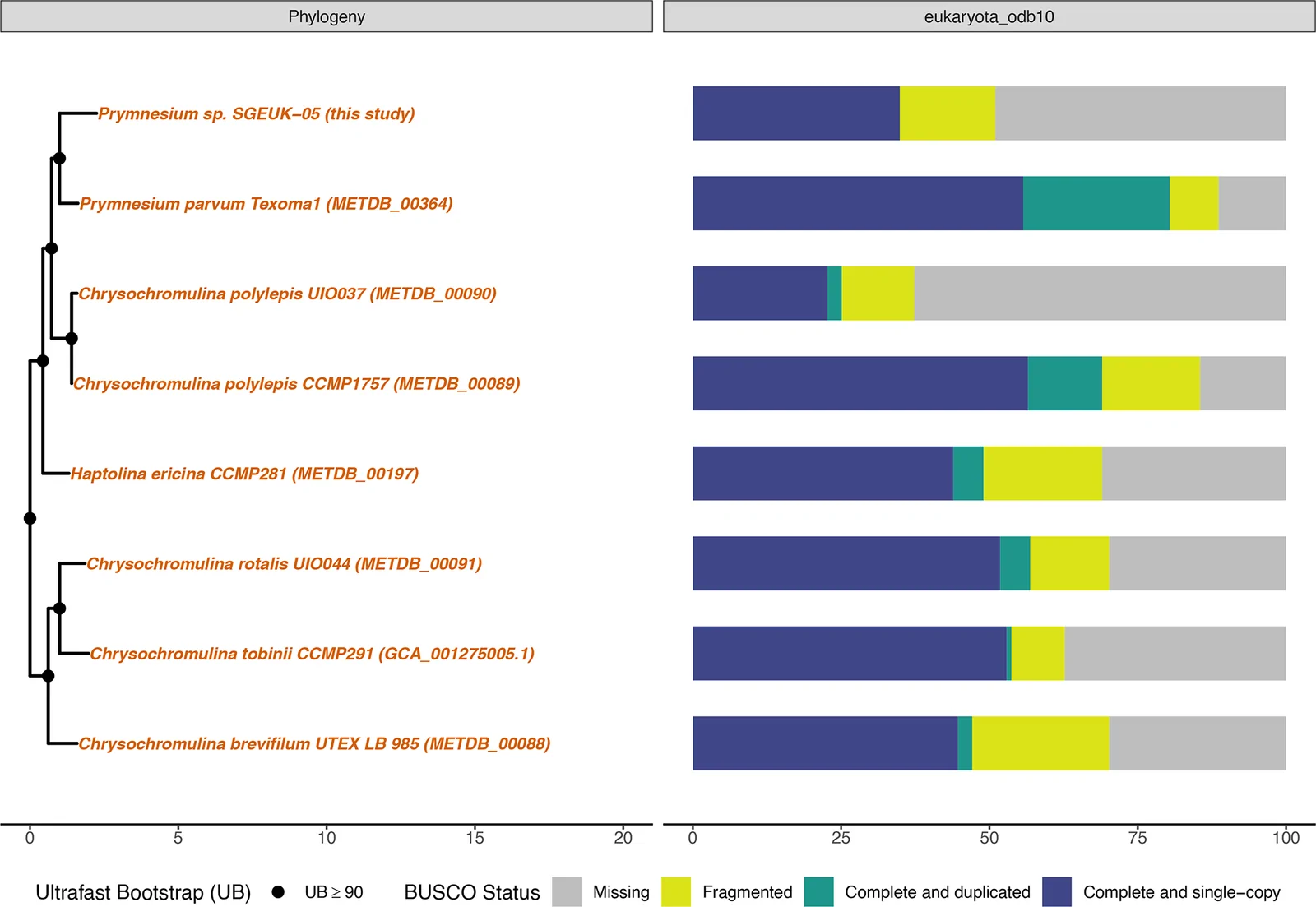
SGEUK-05 is phylogenetically placed in the genus *Prymnesium*. From left to right, first, a maximum-likelihood phylogeny that shows the placement of SGEUK-05 in the haptista tree of life among haptophyte lineages. This tree was generated using IQ-TREE2 on an alignment of BUSCO stramenopiles_odb10 HMMs constructed using the PHYling_unified pipeline. Taxon labels in the phylogeny are shown coloured by their assigned taxonomic family (orange, Prymnesiales). Next, in association with this phylogeny, a bar chart of the BUSCO ‘protein’ completion status for the eukaryota_odb10 set is shown. Bars show the percentage of genes found in each genome annotation as a percentage of the total gene set and are coloured by BUSCO status (missing, grey; fragmented, yellow; complete and duplicated, green; complete and single copy, blue).

### Comparative genomics of SGEUK-03 reveals genes involved in host interaction

We performed OrthoFinder analysis, comparing SGEUK-03 with other fungi with a focus on comparisons to other members of the Lobulomycetales and Chytridiomycota. In total, we identified 75,458 phylogenetically HOGs among all fungi, of which 44,619 were identified among all Chytridiomycota. Of those, 8,192 were identified among the Lobulomycetales, with a total of 4,238 HOGs identified in SGEUK-03. Of the 5,630 genes in SGEUK-03, 4,886 genes (86.8%) were assigned to HOGs. Given that SGEUK-03 represents an incomplete MAG, we focused our analyses on high-copy number orthogroups (≥5 members) in SGEUK-03 and compared these to the average copy numbers in other fungal groups (Fig. 5).

**Fig. 5.**
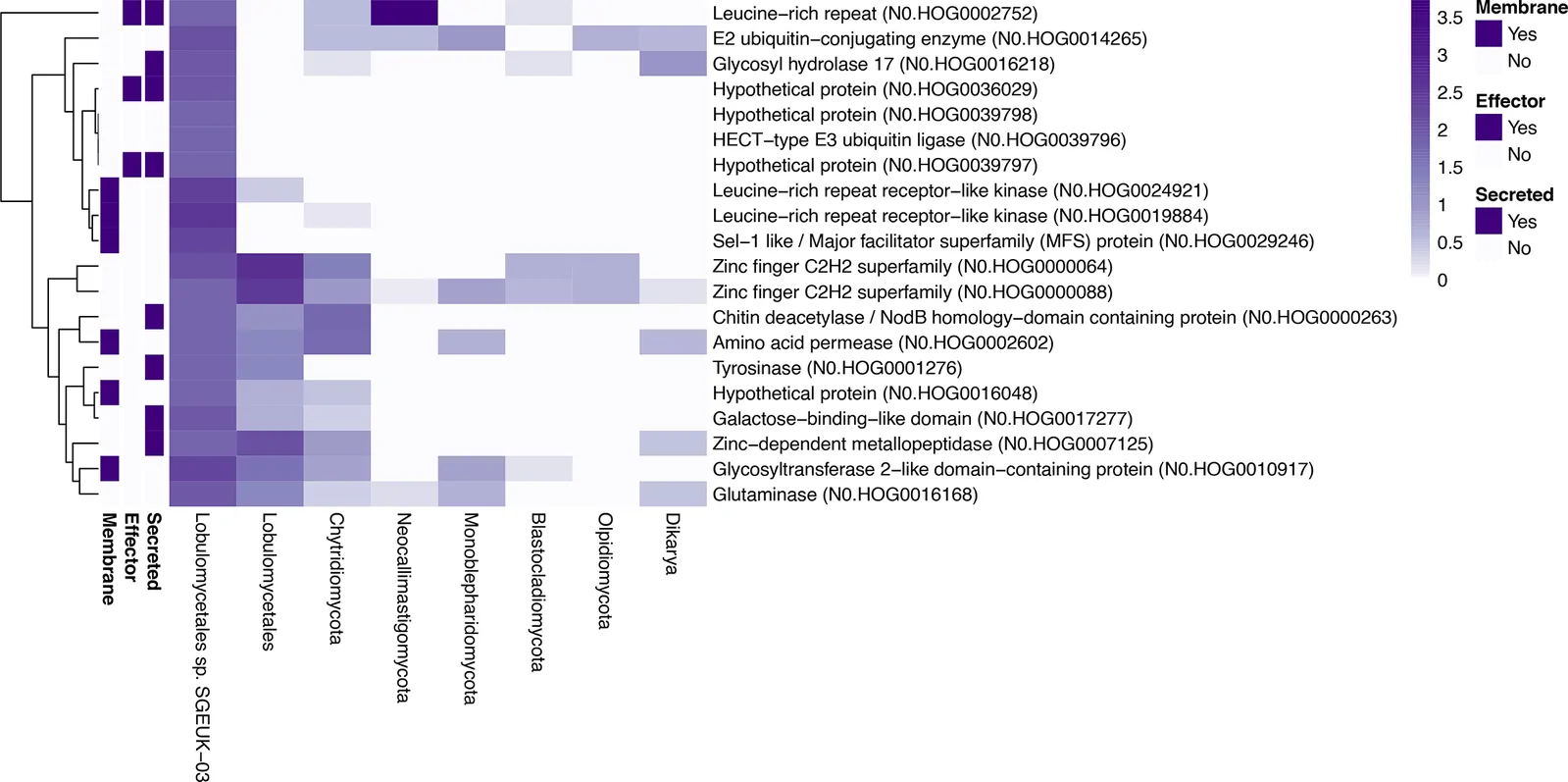
Only some high-copy orthogroups in SGEUK-3 are shared with close relatives. A heatmap is depicted here visualizing the log-scaled copy numbers of phylogenetically HOGs with five or more copies in SGEUK-03 in comparison to the average copy number of the same HOGs in other fungal taxonomic groups (i.e. other members of the order Lobulomycetales, other Chytridiomycota lineages or other fungal phyla). Additionally, whether these genes are predicted in the annotation of SGEUK-03 to be membrane-bound, secreted or as plant-effector is indicated on the left of the heatmap. Further, the dendrogram to the left of the heatmap clusters the HOGs by similarity in log-scaled counts between the different HOGs.

In total, there were 20 high-copy HOGs, of which 5 were unique to SGEUK-03. These included three hypothetical proteins (of which two were predicted cytoplasmic effectors), a Hect-type E3 ubiquitin ligase and a Sel-1 like/Major Facilitator Superfamily (MFS) protein. Six HOGs were shared exclusively with other Chytridiomycota or Lobulomycetales, including genes potentially involved in host interactions, such as leucine-rich repeat receptor-like kinases (LRR-RLKs), a galactose-binding like domain encoding gene, a tyrosinase and a secreted chitin deacetylase (NodB homology-domain containing protein). Additionally, three HOGs were shared with other early-diverging fungal lineages, including a transcription factor, a GT2 and an LRR effector. Finally, six HOGs were shared across distant fungi, including Dikarya, such as GH17, transcription factors and a glutaminase.

## Discussion

### Draft MAGs represent key ‘omics resources of undersequenced eukaryotic lineages

We report the successful recovery of five eukaryotic MAGs associated with the leaves of the seagrass *Z. marina*. The five draft MAGs represent undersequenced eukaryotic lineages including three MAGs representing diatoms in the family Bacillariaceae (93, 70 and 31% BUSCO completeness), one MAG representing a haptophyte algae in the genus *Prymnesium* (40% BUSCO completeness) and a single fungal MAG in the order Lobulomycetales (65% BUSCO completeness). We annotated these five draft eukaryotic MAGs, providing an invaluable functional resource to enable foundational insight into their roles in the marine ecosystem. While all five MAGs represent relatively novel ‘omics resources, we highlight SGEUK-03, which we could only taxonomically place at the order level as part of the Lobulomycetales, a group with only two sequenced genomes, both of which are distant relatives of this MAG. We note that existing BUSCO gene sets are not well suited to early-diverging fungi, such as chytrids, so the reported 65% completeness for SGEUK-03 likely underestimates its true genome completeness and gene content. However, for context, completeness for the other available Lobulomycetales genomes is higher (82–92%; Fig. 2), underscoring that SGEUK-03’s lower completeness likely reflects its status as an incomplete draft MAG. All five of the recovered MAGs also had relatively low sequencing depth (e.g. SGEUK-03 was highest with mean ~7.71× coverage), which likely contributed to the observed assembly fragmentation across all MAGs. Overall, all five eukaryotic MAGs represent significant contributions of genomic novelty, and we expect comparative genomic and functional approaches by the research community to further elucidate their evolution and ecology.

### Diatoms and phytoplankton are critical, yet understudied, primary producers in seagrass ecosystems

Seagrass leaves serve as an ideal habitat and stable refuge for marine diatoms and algae. In fact, diatoms, including members in the Bacillariaceae, make up the biggest component of the microscopic epiphytic biomass associated with seagrass leaves [19, 217–219], have the largest relative abundance within the microbial eukaryotic community associated with *Z. marina* globally [25] and are considered part of *Z. marina*’s core epiphytic leaf community [26, 27]. Similarly, phytoplankton and other microalgae are another important part of the epiphytic community of seagrasses [18, 24, 220]. Both diatoms and microalgae make significant contributions to primary productivity, are seasonally variable and serve as important food sources in seagrass bed ecosystems [19, 20, 218, 221–224]. In this work, seagrass leaves were free of a visible epiphytic biomass layer, suggesting that the recovery of these MAGs might indicate a rather tight association of these taxa with *Z. marina* leaf tissues. We hope that these draft MAGs and their annotations provide useful resources for the community to begin to interrogate their genomics, possible functional roles, community dynamics in seagrass beds and the resilience of these microbial eukaryotes in the face of global climate change.

### Lobulomycetales may have a biotrophic or symbiotic role in seagrass ecosystems

SGEUK-03 clustered within Novel Clade SW-I in the Lobulomycetales (Fig. 3). Known isolated members of Novel Clade SW-I are chytrids that are parasites of freshwater algae and diatoms [203, 204, 225]. While additional unclassified members of Novel Clade SW-I are only known from amplicon-based marine surveys of deep sea sediments, open ocean and seagrass ecosystems [36, 198, 199]. Overall, the Lobulomycetales are a relatively understudied group of chytrid fungi that have been found to span terrestrial, freshwater and marine ecosystems as both parasites and saprobes [43]. Given this diversity and the absence of isolated representatives from seagrass environments, the ecological role of SGEUK-03 remains unclear. However, insights from its genome, specifically its effector and CAZyme profiles (Fig. S1, Table S5), provide clues to its possible functional role.

Effectors are critical for all plant–fungal interactions [226–229] including for symbiotic and biotrophic fungi which still need to evade host plant recognition and suppress host defenses to persist [230, 231]. Additionally, parasitic chytrids of animals and plants have been observed to have a repertoire of effectors proteins, as well as pathogenicity factors [166, 170, 232–234]. SGEUK-03 has a larger number of cytoplasmic effectors than apoplastic effectors, a pattern that might be consistent with an intracellular role. The majority of predicted effectors were hypothetical proteins with limited functional annotation. While the precise function of these effectors remains unclear, their abundance in SGEUK-03 points to a host-associated role.

We can explore the CAZyme diversity of SGEUK-03 to further hone in on its functional role. CAZyme-encoding genes vary among fungal species and likely reflect their adaptation to different nutritional niches [235]. For example, plant pathogens have been found to have, on average, a higher number of CAZymes (mean=464) than saprophytic fungi (mean=338) and animal pathogens (mean=189) [236]. Plant cell wall-degrading enzymes, such as GHs, CEs and PLs, in particular, are often employed by fungal plant pathogens [237]. Necrotrophic pathogens deploy these cell wall-degrading enzymes to promote host damage and colonization [238], whereas endophytic fungi and biotrophs tend to have fewer of these cell wall-degrading enzymes [238–241]. While CAZyme diversity has been widely explored on land, particularly in the Dikaryan fungi, little characterization of these enzymes has been done in chytrids. However, initial reports suggest that these fungi can have a wide range, with estimates from Chytridiomycota ranging from 9 to 442 CAZymes (mean=196) [242].

SGEUK-03 had relatively few CAZymes overall (121 total), below both the Chytridiomycota mean (196) and the reported averages for dikaryon plant pathogens (464) and saprophytic fungi (338), with a notable absence of enzymes reported to be involved in the degradation of plant cell wall components such as cellulose (GH6, GH7, GH12, GH45, GH61, CBM1), hemicellulose (GH10, GH11, GH74, GH27, GH36, GH26, GH43, GH51, GH54, GH62, GH67, GH115) and pectin (GH28, GH78, PL1, PL3, PL4, PL9) [236, 239, 243, 244]. Notably, GH3 and GH35, broad-specificity glycosidases not restricted to plant cell wall degradation, were present but at low copy number (one gene each), consistent with a minimal rather than absent role for general carbohydrate metabolism. Instead, the GTs were the most represented group (53.7% of CAZymes), with GT2 being the most frequent, a pattern perhaps consistent with fungi that modify their own cell wall to evade detection, rather than degrade host cell walls.

Interestingly, the CAZyme profile of SGEUK-03 shares some features with that of the obligate mutualistic symbiont *Rhizophagus irregularis*, an arbuscular mycorrhizal fungus that engages in a mutualistic relationship with land plants [245]. Both fungi show a shared absence of canonical plant cell wall-degrading families (e.g. GH6, GH7, GH12, GH28, GH78, PL1, PL3). The relatively low CAZyme diversity of SGEUK-03, combined with its effector content, suggests that it may engage in a similar strategy of minimal host damage while exploiting host resources. This idea is further supported by the prediction from CATAStrophy [98], which classified SGEUK-03 as having a CAZyme content consistent with a symbiotic lifestyle. However, it is important to note that CATAStrophy was trained on filamentous dikaryon fungi, and its application to chytrids may be limited. Additionally, as the genome of SGEUK-03 is incomplete, we cannot rule out the presence of additional CAZymes, including from plant cell wall-degrading families discussed above, that could alter this classification or the interpretation of SGEUK-03’s CAZyme profile more generally. Regardless, on the whole, the effector and CAZyme profiles of SGEUK-03 may suggest that it has a symbiotic or biotrophic role in seagrass ecosystems.

### Are Lobulomycetales chytrids biotrophic parasites or symbionts of seagrasses themselves?

The frequent identification of Lobulomycetales species in *Z. marina* leaf epiphyte and endophyte samples from previous amplicon surveys [25, 36] supports the hypothesis that SGEUK-03 may be closely associated with *Z. marina*. Microscopic observations of chytrids directly interacting with seagrass tissues further support a possible symbiotic or weakly parasitic role [246]. However, given the diversity of hosts that chytrids can associate with, we cannot rule out the possibility that SGEUK-03 may also be parasitizing a diatom or algal host that is itself associated with *Z. marina*. While we saw no clear coverage pattern (e.g. 1-to-1) which might suggest that one of the other MAGs might be a host for SGEUK-03, another possibility could be that SGEUK-03 is a generalist chytrid, capable of associating with multiple hosts, including *Z. marina* and members of its epiphytic community. We also cannot fully rule out other ecological roles that would not require a close, ongoing host association, including a primarily saprotrophic lifestyle on decaying plant or algal material, transient epiphytic growth on the leaf surface or opportunistic colonization of already-stressed or senescing host tissue; distinguishing among these possibilities will require direct microscopic evidence of interaction with *Z. marina*.

Chytrids are well-known parasites of a broad range of phytoplankton, including diatoms, green algae, dinoflagellates and cyanobacteria, and can display both high host specificity and broad generalist strategies, depending on environmental conditions and host availability [203, 204, 247–255]. A generalist strategy allows for infection of multiple species enabling chytrids to persist despite fluctuations in available host communities, with trade-offs including lower growth rates as a result of needing to maintain the genomic potential to evade diverse host immune responses [251]. Members of the Lobulomycetales and marine fungi more generally have been observed to exhibit some seasonality in their emergence, which has been hypothesized to relate to host dynamics and environmental conditions [44, 256–258]. Blooms of parasitic chytrids, for example, frequently accompany an increased abundance of diatoms that the chytrids infect and ultimately kill. Previous work has suggested that Lobulomycetales sp. associated with seagrasses experience bloom-like dynamics, which could relate to seasonality, environmental conditions or host availability [25, 36]. It is possible that the presence of SGEUK-03 in seagrass ecosystems is similarly influenced by seasonal patterns in host availability, whether that host is seagrass itself or associated epiphytic algae or diatoms. Further work is needed to confirm and explore the host specificity of SGEUK-03.

### SGEUK-03’s high-copy orthologs may represent a functional repertoire focused on host association

To better understand its functional potential, particularly its interactions with its host, we used comparative genomics approaches to examine SGEUK-03’s highest copy orthologs. We found 20 high-copy gene families with functions that indicate they may be involved in host or environmental recognition, evasion of host immune responses and nutrient acquisition.

Among the high-copy orthogroups, we identified two LRR-LRKs, which are receptor kinases that detect external signals and are often implicated in non-self recognition. For example, LRR-LRKs are best known for the role they play in plant and animal innate immunity [259, 260]. Dramatic gene family expansions of LRR-LRKs are observed in plants and animals, but these genes are largely missing in dikaryon fungi, whose innate immune system is still not fully understood, although a small number of LRR-LRKs have been reported in chytrids and zygomycetes [261–263]. The HOGs that included LRR-LRKs were shared with other chytrids and were the most abundant HOGs identified for SGEUK-03. Aquatic chytrids have motile zoospores that enable the targeting of specific hosts and substrates, a capability that is absent in dikaryan spores, which lack flagella [45]. For instance, chytrid zoospores can be attracted to dissolved molecules in the environment which may facilitate host detection [264–266]. We speculate that these LRR-LRKs may be involved in environmental sensing and host detection in SGEUK-03, although a role in innate immunity, similar to that of plants and animals, could also be possible.

A galactose-binding domain protein, another high-copy gene family shared with chytrids, may support early host recognition and adhesion. Galactose is an important sugar in seagrass cell walls [267]. Additionally, a galactose-binding CBM plays an important role in host–fungal interactions through mediating chemotaxis towards the host, attachment and virulence in a chytrid pathogen [268]. SGEUK-03 may be similarly using this gene family for sensing and adhering to host tissues during colonization, establishing an interaction with the host early in the infection process.

In addition to host recognition, SGEUK-03 may possess mechanisms to evade host immune responses during and after invasion. Fungi have evolved several strategies to evade host immune responses including modifying their cell wall composition and secreting effector proteins to protect cell walls or help evade host detection [269, 270]. SGEUK-03 possesses a suite of effectors, including two unique secreted cytoplasmic effector families. In addition, SGEUK-03 encodes a unique family of Hect-type E3 ubiquitin ligases. These ligases play critical roles in regulating protein degradation through modification, which impacts cellular processes such as stress responses, endocytosis, cell cycling and pathogenicity [271–275]. Such effectors and ubiquitin ligases may provide SGEUK-03 with the ability to manipulate host cell machinery and suppress immune responses.

Additionally, SGEUK-03 shares a gene family annotated as a chitin deacetylase (NodB homology-domain containing protein) with other chytrids. Chitin deacetylases can modify the fungal cell wall during plant invasion making it less recognizable to plant defence systems [276, 277]. Chitin deacetylases with NodB homology are also involved in invasion and signalling by symbiotic fungi [278]. SGEUK-03 may similarly modify its cell wall to evade host immunity or maintain host interactions.

SGEUK-03 has a GH family 17 (GH17) enzyme gene family, which it shared with distant fungal lineages, including members of Dikarya. GH17 enzymes are involved in the modification of cell wall glucan in both fungal and plant cells, which is essential for the growth, development and host–pathogen interactions [279–281]. On land, *Cladosporium fulvum*, a hemibiotrophic fungal pathogen of tomatoes, secretes a GH17 which triggers the release of a damage-associated molecular pattern from the host cell wall, triggering host innate immunity and ultimately cell death [282]. Further, GH17 enzymes in fungi are abundant and thought to have important roles in global carbon cycling in the open ocean [283]. For example, a marine *Cladosporium* species secretes GH17 when utilizing laminarin [284], which is an algal and diatom-derived β-glucan that is abundant in the ocean [285]. It is possible that GH17 enzymes in SGEUK-03 could play a similar role in interacting with host cell walls, either during invasion or as part of a defensive strategy.

Tyrosinases, a high-copy gene family of SGEUK-03 shared with other chytrid lineages, have been previously reported as expanded and implicated in host–pathogen interactions [286, 287]. These enzymes are involved in melanin biosynthesis, which contribute to fungal virulence by protecting cells from UV radiation, reactive oxygen species (ROS) and hydrolytic enzymes that degrade fungal cell walls [288–290]. Melanin production could provide SGEUK-03 with protection from host defences, as ROS accumulation is a common plant response to pathogen recognition [238, 291]. Therefore, the putative tyrosinases in SGEUK-03 might provide protection from ROS-mediated host defences, ensuring its survival within host tissues.

We also found that gene families with functions likely related to nutrient acquisition, detoxification and stress responses including several transcription factors and transporter genes. This included a unique Sel-1 like/MFS transporter family, which are a group of transporters vital in cross-membrane transport of organic solutes. They are key in fungal nutrient acquisition and may play roles in detoxification or efflux of harmful plant compounds [292]. Overall, the observed suite of high-copy orthologs in SGEUK-03 supports a functional landscape of gene families with putative annotations related to host or environmental recognition, evasion and interaction.

## Conclusion

We successfully recovered five high-quality draft eukaryotic MAGs from DNA isolated from the leaves of the seagrass *Z. marina*, representing previously undersequenced eukaryotic lineages. These include three MAGs of diatoms from the family Bacillariaceae, one MAG from a haptophyte alga in the genus *Prymnesium* and one fungal MAG from the order Lobulomycetales. The functional annotation of these draft MAGs offers a valuable resource for understanding their ecological roles in the marine environment. Notably, we placed SGEUK-03 within the novel clade SW-I of the Lobulomycetales and performed comparative genomic analysis, suggesting a possible symbiotic or biotrophic lifestyle based on its effector and CAZyme repertoire. Further exploration of high-copy orthogroups in SGEUK-03 revealed a suite of gene families potentially involved in host or environmental interactions, recognition and immune evasion. While additional research is required to clarify host specificity and further characterize these MAGs, our findings contribute to the growing knowledge of marine microbial eukaryotic diversity and provide a genomic foundation for future studies on the ecological roles and evolutionary trajectories of these enigmatic micro-organisms.

## Supplementary material

10.1099/mgen.0.001845 Supplementary Material 1.

10.1099/mgen.0.001845 Supplementary Material 2.

## Abbreviations

CAZyme: carbohydrate-active enzyme
CBM: carbohydrate-binding module
CE: carbohydrate esterase
GH: glycoside hydrolase
GT: glycosyltransferase
HOG: hierarchical orthogroup
LRR-RLK: leucine-rich repeat receptor-like kinase
MAG: metagenome-assembled genome
MFS: Major Facilitator Superfamily
PL: polysaccharide lyase
ROS: reactive oxygen species

## References

[1] Peay KG, Kennedy PG, Talbot JM (2016). Dimensions of biodiversity in the Earth mycobiome. Nat Rev Microbiol.

[2] Amend A, Burgaud G, Cunliffe M, Edgcomb VP, Ettinger CL (2019). Fungi in the marine environment: open questions and unsolved problems. mBio.

[3] Peng X, Amend AS, Baltar F, Blanco‐Bercial L, Breyer E (2024). Planktonic marine fungi: a review. JGR Biogeosci.

[4] Hawksworth DL, Lücking R (2017). Fungal diversity revisited: 2.2 to 3.8 million species. Microbiol Spectr.

[5] Jones EBG (2011). Are there more marine fungi to be described?. Bot Mar.

[6] Jones EBG, Suetrong S, Sakayaroj J, Bahkali AH, Abdel-Wahab MA (2015). Classification of marine Ascomycota, Basidiomycota, Blastocladiomycota and Chytridiomycota. Fungal Divers.

[7] Jones EBG, Pang K-L, Abdel-Wahab MA, Scholz B, Hyde KD (2019). An online resource for marine fungi. Fungal Divers.

[8] Tisthammer KH, Cobian GM, Amend AS (2016). Global biogeography of marine fungi is shaped by the environment. Fungal Ecol.

[9] Morales SE, Biswas A, Herndl GJ, Baltar F (2019). Global structuring of phylogenetic and functional diversity of pelagic fungi by depth and temperature. Front Mar Sci.

[10] Breyer E, Stix C, Kilker S, Roller BRH, Panagou F (2024). The overlooked contribution of pelagic fungi to ocean biomass. *SSRN*.

[11] Breyer E, Zhao Z, Herndl GJ, Baltar F (2022). Global contribution of pelagic fungi to protein degradation in the ocean. Microbiome.

[12] Baltar F, Zhao Z, Herndl GJ (2021). Potential and expression of carbohydrate utilization by marine fungi in the global ocean. Microbiome.

[13] Peng X, Valentine DL (2021). Diversity and N_2_O production potential of fungi in an oceanic oxygen minimum zone. *J Fungi (Basel*).

[14] Hassett BT, Vonnahme TR, Peng X, Jones EBG, Heuzé C (2020). Global diversity and geography of planktonic marine fungi. Bot Mar.

[15] Hemminga MA, Duarte CM (2000). Seagrass Ecology.

[16] Fourqurean JW, Duarte CM, Kennedy H, Marbà N, Holmer M (2012). Seagrass ecosystems as a globally significant carbon stock. Nature Geosci.

[17] Orth RJ, Carruthers TJB, Dennison WC, Duarte CM, Fourqurean JW (2006). A global crisis for seagrass ecosystems. BioScience.

[18] Cummins SP, Roberts DE, Ajani P, Underwood AJ (2004). Comparisons of assemblages of phytoplankton between open water and seagrass habitats in a shallow coastal lagoon. Mar Freshw Res.

[19] Prazukin AV, Lee RI, Firsov YK, Kapranov SV (2022). Vertical distribution of epiphytic diatoms in relation to the eelgrass *Zostera noltii* canopy biomass and height. Aquat Bot.

[20] Lebreton B, Richard P, Galois R, Radenac G, Pfléger C (2011). Trophic importance of diatoms in an intertidal *Zostera noltii* seagrass bed: evidence from stable isotope and fatty acid analyses. Estuar Coast Shelf Sci.

[21] Kolátková V, Mooney M, Kelly K, Hineva E, Gawryluk RMR (2023). Eelgrass (*Zostera* spp.) associated phytomyxids are host-specific congeneric parasites and predominant eukaryotes in the eelgrass rhizosphere on a global scale. Environ Microbiol.

[22] Elliott JK, Simpson H, Teesdale A, Replogle A, Elliott M (2019). A novel phagomyxid parasite produces sporangia in root hair galls of eelgrass (*Zostera marina*). Protist.

[23] Man In’t Veld WA, Karin CH, van Rijswick PCJ, Meffert JP, Boer E (2019). *Multiple halophytophthora* spp. and *Phytophthora* spp. including *P. gemini*, *P. inundata* and *P. chesapeakensis* sp. nov. isolated from the seagrass *Zostera marina* in the northern hemisphere. Eur J Plant Pathol.

[24] Armitage AR, Frankovich TA, Heck KL, Fourqurean JW (2005). Experimental nutrient enrichment causes complex changes in seagrass, microalgae, and macroalgae community structure in Florida Bay. *Estuaries*.

[25] Ettinger CL, Vann LE, Eisen JA (2021). Global diversity and biogeography of the *Zostera marina* mycobiome. Appl Environ Microbiol.

[26] Bengtsson MM, Bühler A, Brauer A, Dahlke S, Schubert H (2017). Eelgrass leaf surface microbiomes are locally variable and highly correlated with epibiotic eukaryotes. Front Microbiol.

[27] Trevizan Segovia B, Sanders-Smith R, Adamczyk EM, Forbes C, Hessing-Lewis M (2021). Microeukaryotic communities associated with the seagrass *Zostera marina* are spatially structured. J Eukaryot Microbiol.

[28] Shoemaker G, Wyllie-Echeverria S (2013). Occurrence of rhizomal endophytes in three temperate northeast pacific seagrasses. Aquatic Botany.

[29] Kirichuk NN, Pivkin MV (2015). Filamentous fungi associated with the seagrass *Zostera marina* Linnaeus, 1753 of Rifovaya Bay (Peter the Great Bay, the Sea of Japan). *Russ J Mar Biol*.

[30] Petersen L-E, Marner M, Labes A, Tasdemir D (2019). Rapid metabolome and bioactivity profiling of fungi associated with the leaf and rhizosphere of the Baltic seagrass *Zostera marina*. Mar Drugs.

[31] Ettinger CL, Eisen JA (2020). Fungi, bacteria and oomycota opportunistically isolated from the seagrass, *Zostera marina*. PLoS One.

[32] Zhang Z-F, Liu F, Liu L-R, Li M, Cai L (2024). Culturing the uncultured marine fungi in the omics age: opportunities and challenges. Fungal Biol Rev.

[33] Hurtado-McCormick V, Kahlke T, Petrou K, Jeffries T, Ralph PJ (2019). Regional and microenvironmental scale characterization of the *Zostera muelleri* seagrass microbiome. Front Microbiol.

[34] Abdel-Wahab MA, Bahkali AH, Elgorban AM, Jones EBG (2021). High-throughput amplicon sequencing of fungi and microbial eukaryotes associated with the seagrass *Halophila stipulacea* (Forssk.) Asch. from Al-Leith mangroves, Saudi Arabia. Mycol Progress.

[35] Quek ZBR, Zahn G, Lee NLY, Ooi JLS, Lee JN (2021). Biogeographic structure of fungal communities in seagrass *Halophilia ovalis* across the Malay Peninsula. Environ Microbiol Rep.

[36] Ettinger CL, Eisen JA (2019). Characterization of the mycobiome of the seagrass, *Zostera marina*, reveals putative associations with marine chytrids. Front Microbiol.

[37] Wainwright BJ, Zahn GL, Arlyza IS, Amend AS (2018). Seagrass‐associated fungal communities follow Wallace’s line, but host genotype does not structure fungal community. J Biogeogr.

[38] Wainwright BJ, Zahn GL, Zushi J, Lee NLY, Ooi JLS (2019). Seagrass-associated fungal communities show distance decay of similarity that has implications for seagrass management and restoration. Ecol Evol.

[39] Trevathan-Tackett SM, Allnutt TR, Sherman CDH, Richardson ME, Crowley TM (2020). Spatial variation of bacterial and fungal communities of estuarine seagrass leaf microbiomes. Aquat Microb Ecol.

[40] Pang K-L, Jones EBG, Abdel-Wahab MA, Adams SJ, Alves A (2023). Recent progress in marine mycological research in different countries, and prospects for future developments worldwide. Bot Mar.

[41] Gonçalves MFM, Esteves AC, Alves A (2022). Marine fungi: opportunities and challenges. *Encyclopedia*.

[42] Hassett BT, Ducluzeau A-LL, Collins RE, Gradinger R (2017). Spatial distribution of aquatic marine fungi across the western Arctic and sub-arctic. Environ Microbiol.

[43] Simmons DR, James TY, Meyer AF, Longcore JE (2009). Lobulomycetales, a new order in the Chytridiomycota. Mycol Res.

[44] Longcore JE (1992). Morphology and zoospore ultrastructure of *Chytriomyces angularis* sp. nov. (Chytridiales). Mycologia.

[45] Laundon D, Cunliffe M (2021). A call for a better understanding of aquatic chytrid biology. Front Fungal Biol.

[46] Sharon I, Morowitz MJ, Thomas BC, Costello EK, Relman DA (2013). Time series community genomics analysis reveals rapid shifts in bacterial species, strains, and phage during infant gut colonization. Genome Res.

[47] Albertsen M, Hugenholtz P, Skarshewski A, Nielsen KL, Tyson GW (2013). Genome sequences of rare, uncultured bacteria obtained by differential coverage binning of multiple metagenomes. Nat Biotechnol.

[48] Bushnell B (2022). BBTools. https://sourceforge.net/projects/bbmap/.

[49] Ma X, Olsen JL, Reusch TBH, Procaccini G, Kudrna D (2021). Improved chromosome-level genome assembly and annotation of the seagrass, *Zostera marina* (eelgrass). F1000Res.

[50] Langmead B, Trapnell C, Pop M, Salzberg SL (2009). Ultrafast and memory-efficient alignment of short DNA sequences to the human genome. Genome Biol.

[51] Li H, Handsaker B, Wysoker A, Fennell T, Ruan J (2009). The sequence alignment/map format and SAMtools. Bioinformatics.

[52] Li D, Liu C-M, Luo R, Sadakane K, Lam T-W (2015). MEGAHIT: an ultra-fast single-node solution for large and complex metagenomics assembly via succinct *de Bruijn* graph. Bioinformatics.

[53] Pronk LJU, Medema MH (2022). Whokaryote: distinguishing eukaryotic and prokaryotic contigs in metagenomes based on gene structure. Microb Genom.

[54] Karlicki M, Antonowicz S, Karnkowska A (2022). Tiara: deep learning-based classification system for eukaryotic sequences. Bioinformatics.

[55] Eren AM, Esen ÖC, Quince C, Vineis JH, Morrison HG (2015). Anvi’o: an advanced analysis and visualization platform for 'omics data. PeerJ.

[56] Hyatt D, Chen G-L, Locascio PF, Land ML, Larimer FW (2010). Prodigal: prokaryotic gene recognition and translation initiation site identification. BMC Bioinformatics.

[57] Lee MD (2019). GToTree: a user-friendly workflow for phylogenomics. Bioinformatics.

[58] Delmont T (2018). Assessing the completion of eukaryotic bins with anvi’o. http://merenlab.org/delmont-euk-scgs.

[59] Eddy SR (2011). Accelerated profile HMM searches. PLoS Comput Biol.

[60] Seemann T barrnap: basic rapid ribosomal RNA predictor. https://github.com/tseemann/barrnap.

[61] Menzel P, Ng KL, Krogh A (2016). Fast and sensitive taxonomic classification for metagenomics with Kaiju. Nat Commun.

[62] Alneberg J, Bjarnason BS, de Bruijn I, Schirmer M, Quick J (2014). Binning metagenomic contigs by coverage and composition. Nat Methods.

[63] Kang DD, Li F, Kirton E, Thomas A, Egan R (2019). MetaBAT 2: an adaptive binning algorithm for robust and efficient genome reconstruction from metagenome assemblies. PeerJ.

[64] Graham ED, Heidelberg JF, Tully BJ (2017). BinSanity: unsupervised clustering of environmental microbial assemblies using coverage and affinity propagation. PeerJ.

[65] Wu Y-W, Tang Y-H, Tringe SG, Simmons BA, Singer SW (2014). MaxBin: an automated binning method to recover individual genomes from metagenomes using an expectation-maximization algorithm. Microbiome.

[66] Saary P, Mitchell AL, Finn RD (2020). Estimating the quality of eukaryotic genomes recovered from metagenomic analysis with EukCC. Genome Biol.

[67] Olm MR, Brown CT, Brooks B, Banfield JF (2017). dRep: a tool for fast and accurate genomic comparisons that enables improved genome recovery from metagenomes through de-replication. ISME J.

[68] Alexander H, Hu SK, Krinos AI, Pachiadaki M, Tully BJ (2023). Eukaryotic genomes from a global metagenomic data set illuminate trophic modes and biogeography of ocean plankton. mBio.

[69] Challis R, Richards E, Rajan J, Cochrane G, Blaxter M (2020). BlobToolKit – interactive quality assessment of genome assemblies. G3 Genes Genom Genet.

[70] Buchfink B, Reuter K, Drost H-G (2021). Sensitive protein alignments at tree-of-life scale using DIAMOND. Nat Methods.

[71] Camacho C, Coulouris G, Avagyan V, Ma N, Papadopoulos J (2009). BLAST+: architecture and applications. BMC Bioinformatics.

[72] Li H (2018). Minimap2: pairwise alignment for nucleotide sequences. Bioinformatics.

[73] Krinos AI, Hu SK, Cohen NR, Alexander H (2021). EUKulele: taxonomic annotation of the unsung eukaryotic microbes. JOSS.

[74] Simão FA, Waterhouse RM, Ioannidis P, Kriventseva EV, Zdobnov EM (2015). BUSCO: assessing genome assembly and annotation completeness with single-copy orthologs. Bioinformatics.

[75] Stajich J, Palmer J (2021). stajichlab/AAFTF: v0.4.1.

[76] Flynn JM, Hubley R, Goubert C, Rosen J, Clark AG (2020). RepeatModeler2 for automated genomic discovery of transposable element families. Proc Natl Acad Sci U S A.

[77] Smit AFA, Hubley R, RepeatMasker GP RepeatMasker. 2013-2015. http://www.repeatmasker.org.

[78] Palmer JM, Stajich J (2020).

[79] Stanke M, Keller O, Gunduz I, Hayes A, Waack S (2006). AUGUSTUS: ab initio prediction of alternative transcripts. Nucleic Acids Res.

[80] Ter-Hovhannisyan V, Lomsadze A, Chernoff YO, Borodovsky M (2008). Gene prediction in novel fungal genomes using an ab initio algorithm with unsupervised training. Genome Res.

[81] Majoros WH, Pertea M, Salzberg SL (2004). TigrScan and GlimmerHMM: two open source ab initio eukaryotic gene-finders. Bioinformatics.

[82] Korf I (2004). Gene finding in novel genomes. BMC Bioinformatics.

[83] Testa AC, Hane JK, Ellwood SR, Oliver RP (2015). CodingQuarry: highly accurate hidden Markov model gene prediction in fungal genomes using RNA-seq transcripts. BMC Genomics.

[84] Haas BJ, Salzberg SL, Zhu W, Pertea M, Allen JE (2008). Automated eukaryotic gene structure annotation using EVidenceModeler and the program to assemble spliced alignments. Genome Biol.

[85] Lowe TM, Eddy SR (1997). tRNAscan-SE: a program for improved detection of transfer RNA genes in genomic sequence. Nucleic Acids Res.

[86] Finn RD, Bateman A, Clements J, Coggill P, Eberhardt RY (2014). Pfam: the protein families database. Nucl Acids Res.

[87] Lombard V, Golaconda Ramulu H, Drula E, Coutinho PM, Henrissat B (2014). The carbohydrate-active enzymes database (CAZy) in 2013. Nucleic Acids Res.

[88] Huang L, Zhang H, Wu P, Entwistle S, Li X (2018). dbCAN-seq: a database of carbohydrate-active enzyme (CAZyme) sequence and annotation. Nucleic Acids Res.

[89] Rawlings ND, Barrett AJ, Bateman A (2014). Using the MEROPS database for proteolytic enzymes and their inhibitors and substrates. Curr Protoc Bioinformatics.

[90] Huerta-Cepas J, Szklarczyk D, Forslund K, Cook H, Heller D (2016). eggNOG 4.5: a hierarchical orthology framework with improved functional annotations for eukaryotic, prokaryotic and viral sequences. Nucleic Acids Res.

[91] Jones P, Binns D, Chang H-Y, Fraser M, Li W (2014). InterProScan 5: genome-scale protein function classification. Bioinformatics.

[92] Boutet E, Lieberherr D, Tognolli M, Schneider M, Bansal P (2016). UniProtKB/Swiss-prot, the manually annotated section of the UniProt KnowledgeBase: how to use the entry view. Methods Mol Biol.

[93] Buchfink B, Xie C, Huson DH (2015). Fast and sensitive protein alignment using DIAMOND. Nat Methods.

[94] Käll L, Krogh A, Sonnhammer ELL (2004). A combined transmembrane topology and signal peptide prediction method. J Mol Biol.

[95] Almagro Armenteros JJ, Tsirigos KD, Sønderby CK, Petersen TN, Winther O (2019). SignalP 5.0 improves signal peptide predictions using deep neural networks. Nat Biotechnol.

[96] Medema MH, Blin K, Cimermancic P, de Jager V, Zakrzewski P (2011). antiSMASH: rapid identification, annotation and analysis of secondary metabolite biosynthesis gene clusters in bacterial and fungal genome sequences. Nucleic Acids Res.

[97] Sperschneider J, Dodds PN (2022). EffectorP 3.0: prediction of apoplastic and cytoplasmic effectors in fungi and oomycetes. Mol Plant Microbe Interact.

[98] Hane JK, Paxman J, Jones DAB, Oliver RP, de Wit P (2019). “CATAStrophy,” a genome-informed trophic classification of filamentous plant pathogens - how many different types of filamentous plant pathogens are there?. Front Microbiol.

[99] Johnson LK, Alexander H, Brown CT (2019). Re-assembly, quality evaluation, and annotation of 678 microbial eukaryotic reference transcriptomes. Gigascience.

[100] Keeling PJ, Burki F, Wilcox HM, Allam B, Allen EE (2014). The Marine Microbial Eukaryote Transcriptome Sequencing Project (MMETSP): illuminating the functional diversity of eukaryotic life in the oceans through transcriptome sequencing. PLoS Biol.

[101] Niang G, Hoebeke M, Meng A, Liu X, Scheremetjew M (2020). METdb: a genomic reference database for marine species. F1000Res.

[102] Scott P, Taylor P, Gardner J, Puértolas A, Panda P (2019). *Phytophthora aleatoria* sp. nov., associated with root and collar damage on *Pinus radiata* from nurseries and plantations. *Australasian Plant Pathol*.

[103] Wawrzyniak I, Courtine D, Osman M, Hubans-Pierlot C, Cian A (2015). Draft genome sequence of the intestinal parasite Blastocystis subtype 4-isolate WR1. Genom Data.

[104] Sambles C, Schlenzig A, O’Neill P, Grant M, Studholme DJ (2015). Draft genome sequences of *Phytophthora kernoviae* and *Phytophthora ramorum* lineage EU2 from Scotland. Genomics Data.

[105] Studholme DJ, McDougal RL, Sambles C, Hansen E, Hardy G (2016). Genome sequences of six *Phytophthora* species associated with forests in New Zealand. Genomics Data.

[106] Minardi D, Studholme DJ, van der Giezen M, Pretto T, Oidtmann B (2018). New genotyping method for the causative agent of crayfish plague (*Aphanomyces astaci*) based on whole genome data. J Invertebr Pathol.

[107] Dittami SM, Corre E, Brillet-Guéguen L, Lipinska AP, Pontoizeau N (2020). The genome of *Ectocarpus subulatus* - a highly stress-tolerant brown alga. Mar Genomics.

[108] Kiselev A, San Clemente H, Camborde L, Dumas B, Gaulin E (2022). A comprehensive assessment of the secretome responsible for host adaptation of the legume root pathogen *Aphanomyces euteiches*. J Fungi (Basel).

[109] Armitage AD, Lysøe E, Nellist CF, Lewis LA, Cano LM (2018). Bioinformatic characterisation of the effector repertoire of the strawberry pathogen *Phytophthora cactorum*. PLoS ONE.

[110] Studholme DJ, Panda P, Sanfuentes Von Stowasser E, González M, Hill R (2019). Genome sequencing of oomycete isolates from Chile supports the New Zealand origin of *Phytophthora kernoviae* and makes available the first *Nothophytophthora* sp. genome. Mol Plant Pathol.

[111] Gann ER, Truchon AR, Papoulis SE, Dyhrman ST, Gobler CJ (2022). *Aureococcus anophagefferens* (Pelagophyceae) genomes improve evaluation of nutrient acquisition strategies involved in brown tide dynamics. J Phycol.

[112] Adams TM, Armitage AD, Sobczyk MK, Bates HJ, Tabima JF (2020). Genomic investigation of the strawberry pathogen *Phytophthora fragariae* indicates pathogenicity is associated with transcriptional variation in three key races. Front Microbiol.

[113] Gaulin E, Pel MJC, Camborde L, San-Clemente H, Courbier S (2018). Genomics analysis of *Aphanomyces* spp. identifies a new class of oomycete effector associated with host adaptation. BMC Biol.

[114] Bowler C, Allen AE, Badger JH, Grimwood J, Jabbari K (2008). The Phaeodactylum genome reveals the evolutionary history of diatom genomes. Nature.

[115] Haas BJ, Kamoun S, Zody MC, Jiang RHY, Handsaker RE (2009). Genome sequence and analysis of the Irish potato famine pathogen Phytophthora infestans. Nature.

[116] Cock JM, Sterck L, Rouzé P, Scornet D, Allen AE (2010). The Ectocarpus genome and the independent evolution of multicellularity in brown algae. Nature.

[117] Mock T, Otillar RP, Strauss J, McMullan M, Paajanen P (2017). Evolutionary genomics of the cold-adapted diatom *Fragilariopsis cylindrus*. Nature.

[118] Radakovits R, Jinkerson RE, Fuerstenberg SI, Tae H, Settlage RE (2012). Draft genome sequence and genetic transformation of the oleaginous alga *Nannochloropis gaditana*. Nat Commun.

[119] Osuna-Cruz CM, Bilcke G, Vancaester E, De Decker S, Bones AM (2020). The *Seminavis robusta* genome provides insights into the evolutionary adaptations of benthic diatoms. Nat Commun.

[120] Hackl T, Martin R, Barenhoff K, Duponchel S, Heider D (2020). Four high-quality draft genome assemblies of the marine heterotrophic nanoflagellate *Cafeteria roenbergensis*. Sci Data.

[121] Yang M, Duan S, Mei X, Huang H, Chen W (2018). The Phytophthora cactorum genome provides insights into the adaptation to host defense compounds and fungicides. Sci Rep.

[122] Hongo Y, Kimura K, Takaki Y, Yoshida Y, Baba S (2021). The genome of the diatom *Chaetoceros tenuissimus* carries an ancient integrated fragment of an extant virus. Sci Rep.

[123] Oliver A, Podell S, Pinowska A, Traller JC, Smith SR (2021). Diploid genomic architecture of *Nitzschia inconspicua*, an elite biomass production diatom. Sci Rep.

[124] Davison JR, Rajwani R, Zhao G, Bewley CA (2023). The genome of antibiotic-producing colonies of the Pelagophyte alga *Chrysophaeum taylorii* reveals a diverse and non-canonical capacity for secondary metabolism. Sci Rep.

[125] Guérin N, Ciccarella M, Flamant E, Frémont P, Mangenot S (2022). Genomic adaptation of the picoeukaryote *Pelagomonas calceolata* to iron-poor oceans revealed by a chromosome-scale genome sequence. Commun Biol.

[126] Makkonen J, Vesterbacka A, Martin F, Jussila J, Diéguez-Uribeondo J (2016). Mitochondrial genomes and comparative genomics of *Aphanomyces astaci* and *Aphanomyces invadans*. Sci Rep.

[127] Gobler CJ, Berry DL, Dyhrman ST, Wilhelm SW, Salamov A (2011). Niche of harmful alga *Aureococcus anophagefferens* revealed through ecogenomics. Proc Natl Acad Sci U S A.

[128] Suzuki S, Ota S, Yamagishi T, Tuji A, Yamaguchi H (2022). Rapid transcriptomic and physiological changes in the freshwater pennate diatom *Mayamaea pseudoterrestris* in response to copper exposure. DNA Res.

[129] Fletcher K, Martin F, Isakeit T, Cavanaugh K, Magill C (2023). The genome of the oomycete *Peronosclerospora sorghi*, a cosmopolitan pathogen of maize and sorghum, is inflated with dispersed pseudogenes. G3 (Bethesda).

[130] Misner I, Blouin N, Leonard G, Richards TA, Lane CE (2014). The secreted proteins of *Achlya hypogyna* and *Thraustotheca clavata* identify the ancestral oomycete secretome and reveal gene acquisitions by horizontal gene transfer. Genome Biol Evol.

[131] Ali SS, Shao J, Lary DJ, Kronmiller B, Shen D (2017). *Phytophthora megakarya* and *P. palmivora*, closely related causal agents of cacao black pod rot, underwent increases in genome sizes and gene numbers by different mechanisms. Genome Biol Evol.

[132] Corteggiani Carpinelli E, Telatin A, Vitulo N, Forcato C, D’Angelo M (2014). Chromosome scale genome assembly and transcriptome profiling of *Nannochloropsis gaditana* in nitrogen depletion. Molecular Plant.

[133] Thorpe P, Vetukuri RR, Hedley PE, Morris J, Whisson MA (2021). Draft genome assemblies for tree pathogens *Phytophthora pseudosyringae* and *Phytophthora boehmeriae*. G3 (Bethesda).

[134] Lee Y, Cho K-S, Seo J-H, Sohn KH, Prokchorchik M (2020). Improved genome sequence and gene annotation resource for the potato late blight pathogen *Phytophthora infestans*. Mol Plant Microbe Interact.

[135] Fletcher K, Shin O-H, Clark KJ, Feng C, Putman AI (2022). Ancestral chromosomes for family Peronosporaceae inferred from a telomere-to-telomere genome assembly of *Peronospora effusa*. Mol Plant Microbe Interact.

[136] Carleson NC, Press CM, High-Quality GNJ (2022). Phased genomes of *Phytophthora ramorum* clonal lineages NA1 and EU1. Mol Plant Microbe Interact.

[137] Botkin J, Chanda AK, Martin FN, Hirsch CD (2022). A reference genome sequence resource for the sugar beet root rot pathogen *Aphanomyces cochlioides*. Mol Plant Microbe Interact.

[138] Tanaka T, Maeda Y, Veluchamy A, Tanaka M, Abida H (2015). Oil accumulation by the oleaginous diatom *Fistulifera solaris* as revealed by the genome and transcriptome. Plant Cell.

[139] Basu S, Patil S, Mapleson D, Russo MT, Vitale L (2017). Finding a partner in the ocean: molecular and evolutionary bases of the response to sexual cues in a planktonic diatom. New Phytologist.

[140] Armbrust EV, Berges JA, Bowler C, Green BR, Martinez D (2004). The genome of the diatom *Thalassiosira pseudonana*: ecology, evolution, and metabolism. Science.

[141] Tyler BM, Tripathy S, Zhang X, Dehal P, Jiang RHY (2006). Phytophthora genome sequences uncover evolutionary origins and mechanisms of pathogenesis. Science.

[142] Rujirawat T, Patumcharoenpol P, Lohnoo T, Yingyong W, Lerksuthirat T (2015). Draft genome sequence of the pathogenic oomycete *Pythium insidiosum* strain Pi-S, isolated from a patient with pythiosis. Genome Announc.

[143] Zepernick BN, Truchon AR, Gann ER, Wilhelm SW (2022). Draft genome sequence of the freshwater diatom *Fragilaria crotonensis* SAG 28.96. Microbiol Resour Announc.

[144] Stajich JE, Vu AL, Judelson HS, Vogel GM, Gore MA (2021). High-quality reference genome sequence for the oomycete vegetable pathogen *Phytophthora capsici* strain LT1534. Microbiol Resour Announc.

[145] Mahan KM, Polle JEW, McKie-Krisberg Z, Lipzen A, Kuo A (2021). Annotated genome sequence of the high-biomass-producing yellow-green alga *Tribonema minus*. Microbiol Resour Announc.

[146] Reghu RJ, Chellappan BV, Beena SH, Sasi A, Vasudevan SE (2020). Draft genome sequence of the oomycete *Globisporangium splendens* strain rgcb-1. Microbiol Resour Announc.

[147] Morgan WR, Tartar A (2023). Decontamination and annotation of the draft genome sequence of the oomycete *Lagenidium giganteum* ARSEF 373. Microbiol Resour Announc.

[148] Seddiki K, Godart F, Aiese Cigliano R, Sanseverino W, Barakat M (2018). Sequencing, *De Novo* assembly, and annotation of the complete genome of a new thraustochytrid species, strain CCAP_4062/3. Genome Announc.

[149] Ohan JA, Hovde BT, Zhang XL, Davenport KW, Chertkov O (2019). Nuclear genome assembly of the microalga *Nannochloropsis salina* CCMP1776. Microbiol Resour Announc.

[150] Lommer M, Specht M, Roy A-S, Kraemer L, Andreson R (2012). Genome and low-iron response of an oceanic diatom adapted to chronic iron limitation. Genome Biol.

[151] Sharma R, Xia X, Cano LM, Evangelisti E, Kemen E (2015). Genome analyses of the sunflower pathogen *Plasmopara halstedii* provide insights into effector evolution in downy mildews and *Phytophthora*. BMC Genomics.

[152] Fletcher K, Klosterman SJ, Derevnina L, Martin F, Bertier LD (2018). Comparative genomics of downy mildews reveals potential adaptations to biotrophy. BMC Genomics.

[153] Engelbrecht J, Duong TA, Prabhu SA, Seedat M, van den Berg N (2021). Genome of the destructive oomycete *Phytophthora cinnamomi* provides insights into its pathogenicity and adaptive potential. BMC Genomics.

[154] Fletcher K, Zhang L, Gil J, Han R, Cavanaugh K (2021). AFLAP: assembly-free linkage analysis pipeline using k-mers from genome sequencing data. Genome Biol.

[155] Liu H, Ma X, Yu H, Fang D, Li Y (2016). Genomes and virulence difference between two physiological races of *Phytophthora nicotianae*. Gigascience.

[156] Denoeud F, Roussel M, Noel B, Wawrzyniak I, Da Silva C (2011). Genome sequence of the stramenopile Blastocystis, a human anaerobic parasite. Genome Biol.

[157] Krajaejun T, Patumcharoenpol P, Rujirawat T, Kittichotirat W, Tangphatsornruang S (2023). MGI short-read genome assemblies of *Pythium insidiosum* (reclassified as *Pythium periculosum*) strains Pi057C3 and Pi050C3. BMC Res Notes.

[158] Jiang RHY, de Bruijn I, Haas BJ, Belmonte R, Löbach L (2013). Distinctive expansion of potential virulence genes in the genome of the oomycete fish pathogen *Saprolegnia parasitica*. PLoS Genet.

[159] Matson MEH, Liang Q, Lonardi S, Judelson HS (2022). Karyotype variation, spontaneous genome rearrangements affecting chemical insensitivity, and expression level polymorphisms in the plant pathogen *Phytophthora* infestans revealed using its first chromosome-scale assembly. PLoS Pathog.

[160] Gentekaki E, Curtis BA, Stairs CW, Klimeš V, Eliáš M (2017). Extreme genome diversity in the hyper-prevalent parasitic eukaryote Blastocystis. PLoS Biol.

[161] Faure C, Veyssière M, Boëlle B, San Clemente H, Bouchez O (2020). Long-read genome sequence of the sugar beet rhizosphere mycoparasite *Pythium oligandrum*. G3 Genes Genom Genet.

[162] Dorrell RG, Kuo A, Füssy Z, Richardson EH, Salamov A (2023). Convergent evolution and horizontal gene transfer in Arctic Ocean microalgae. Life Sci Alliance.

[163] Mohammadi M, Smith EA, Stanghellini ME, Kaundal R (2021). Insights into the host specificity of a new oomycete root pathogen, *Pythium brassicum* P1: whole genome sequencing and comparative analysis reveals contracted regulation of metabolism, protein families, and distinct pathogenicity repertoire. Int J Mol Sci.

[164] McMullan M, Gardiner A, Bailey K, Kemen E, Ward BJ (2015). Evidence for suppression of immunity as a driver for genomic introgressions and host range expansion in races of *Albugo candida*, a generalist parasite. *eLife*.

[165] Kämper J, Kahmann R, Bölker M, Ma L-J, Brefort T (2006). Insights from the genome of the biotrophic fungal plant pathogen *Ustilago maydis*. Nature.

[166] Farrer RA, Martel A, Verbrugghe E, Abouelleil A, Ducatelle R (2017). Genomic innovations linked to infection strategies across emerging pathogenic chytrid fungi. Nat Commun.

[167] Mondo SJ, Dannebaum RO, Kuo RC, Louie KB, Bewick AJ (2017). Widespread adenine N6-methylation of active genes in fungi. Nat Genet.

[168] Haitjema CH, Gilmore SP, Henske JK, Solomon KV, de Groot R (2017). A parts list for fungal cellulosomes revealed by comparative genomics. Nat Microbiol.

[169] Ahrendt SR, Quandt CA, Ciobanu D, Clum A, Salamov A (2018). Leveraging single-cell genomics to expand the fungal tree of life. Nat Microbiol.

[170] van de Vossenberg BTLH, Warris S, Nguyen HDT, van Gent-Pelzer MPE, Joly DL (2019). Comparative genomics of chytrid fungi reveal insights into the obligate biotrophic and pathogenic lifestyle of *Synchytrium endobioticum*. Sci Rep.

[171] Chang Y, Rochon D, Sekimoto S, Wang Y, Chovatia M (2021). Genome-scale phylogenetic analyses confirm Olpidium as the closest living zoosporic fungus to the non-flagellated, terrestrial fungi. Sci Rep.

[172] Amses KR, Simmons DR, Longcore JE, Mondo SJ, Seto K (2022). Diploid-dominant life cycles characterize the early evolution of Fungi. Proc Natl Acad Sci USA.

[173] Ettinger CL, Ostovar T, Yacoub M, Ahrendt S, Hice RH (2023). Genomes and transcriptomes help unravel the complex life cycle of the blastoclad fungus, *Coelomomyces lativittatus*, an obligate parasite of mosquitoes and microcrustaceans. Mycologia.

[174] Leonard G, Galindo LJ, Milner DS, Avelar GM, Gomes-Vieira AL (2022). A genome sequence assembly of the phototactic and optogenetic model fungus *Blastocladiella emersonii* reveals a diversified nucleotide-cyclase repertoire. Genome Biol Evol.

[175] Chang Y, Wang S, Sekimoto S, Aerts AL, Choi C (2015). Phylogenomic analyses indicate that early fungi evolved digesting cell walls of algal ancestors of land plants. Genome Biol Evol.

[176] Gómez Luciano LB, Tsai IJ, Chuma I, Tosa Y, Chen Y-H (2019). Blast fungal genomes show frequent chromosomal changes, gene gains and losses, and effector gene turnover. Mol Biol Evol.

[177] Clemons R, Yacoub M, Faust E, Toledo LF, Jenkinson TS Discovery of an endogenous DNA virus in the amphibian killing fungus and its association with pathogen genotype and virulence. Microbiology.

[178] Cuomo CA, Güldener U, Xu J-R, Trail F, Turgeon BG (2007). The *Fusarium graminearum* genome reveals a link between localized polymorphism and pathogen specialization. Science.

[179] Youssef NH, Couger MB, Struchtemeyer CG, Liggenstoffer AS, Prade RA (2013). The genome of the anaerobic fungus *Orpinomyces* sp. strain C1A reveals the unique evolutionary history of a remarkable plant biomass degrader. Appl Environ Microbiol.

[180] Russ C, Lang BF, Chen Z, Gujja S, Shea T (2016). Genome sequence of *Spizellomyces punctatus*. Genome Announc.

[181] Wilken StE, Monk JM, Leggieri PA, Lawson CE, Lankiewicz TS (2021). Experimentally validated reconstruction and analysis of a genome-scale metabolic model of an anaerobic neocallimastigomycota fungus. mSystems.

[182] Brown JL, Swift CL, Mondo SJ, Seppala S, Salamov A (2021). Co‑cultivation of the anaerobic fungus *Caecomyces churrovis* with *Methanobacterium bryantii* enhances transcription of carbohydrate binding modules, dockerins, and pyruvate formate lyases on specific substrates. Biotechnol Biofuels.

[183] Goodwin SB, Ben M’Barek S, Dhillon B, Wittenberg AHJ, Crane CF (2011). Finished genome of the fungal wheat pathogen *Mycosphaerella graminicola* reveals dispensome structure, chromosome plasticity, and stealth pathogenesis. PLoS Genet.

[184] Joneson S, Stajich JE, Shiu S-H, Rosenblum EB (2011). Genomic transition to pathogenicity in chytrid fungi. PLoS Pathog.

[185] Li Y, Li Y, Jin W, Sharpton TJ, Mackie RI (2019). Combined genomic, transcriptomic, proteomic, and physiological characterization of the growth of *Pecoramyces* sp. F1 in monoculture and co-culture with a syntrophic methanogen. Front Microbiol.

[186] Ettinger CL, Eisen JA, Stajich JE (2024). Genomic streamlining of seagrass-associated *Colletotrichum* sp. may be related to its adaptation to a marine monocot host. Microbiology.

[187] Read BA, Kegel J, Klute MJ, Kuo A, Lefebvre SC (2013). Pan genome of the phytoplankton Emiliania underpins its global distribution. Nature.

[188] Hulatt CJ, Wijffels RH, Posewitz MC (2021). The genome of the haptophyte *Diacronema lutheri* (*Pavlova lutheri*, Pavlovales): a model for lipid biosynthesis in eukaryotic algae. Genome Biol Evol.

[189] Hovde BT, Deodato CR, Hunsperger HM, Ryken SA, Yost W (2015). Genome sequence and transcriptome analyses of chrysochromulina tobin: metabolic tools for enhanced algal fitness in the prominent order Prymnesiales (*Haptophyceae*). PLoS Genet.

[190] Steenwyk JL, Buida TJ, Li Y, Shen X-X, Rokas A (2020). ClipKIT: a multiple sequence alignment trimming software for accurate phylogenomic inference. *PLoS Biol*.

[191] Minh BQ, Schmidt HA, Chernomor O, Schrempf D, Woodhams MD (2020). IQ-TREE 2: new models and efficient methods for phylogenetic inference in the genomic era. Mol Biol Evol.

[192] Chernomor O, von Haeseler A, Minh BQ (2016). Terrace aware data structure for phylogenomic inference from supermatrices. Syst Biol.

[193] Kalyaanamoorthy S, Minh BQ, Wong TKF, von Haeseler A, Jermiin LS (2017). ModelFinder: fast model selection for accurate phylogenetic estimates. Nat Methods.

[194] The R project for statistical computing. https://www.r-project.org/.

[195] Yu G, Smith DK, Zhu H, Guan Y, Lam T-Y (2017). Ggtree: an r package for visualization and annotation of phylogenetic trees with their covariates and other associated data. Methods Ecol Evol.

[196] Mise K, Iwasaki W (2022). Unexpected absence of ribosomal protein genes from metagenome-assembled genomes. ISME Commun.

[197] Gruber-Vodicka HR, Seah BKB, Pruesse E (2020). phyloFlash: rapid small-subunit rRNA profiling and targeted assembly from metagenomes. mSystems.

[198] Majaneva M, Rintala J-M, Piisilä M, Fewer DP, Blomster J (2012). Comparison of wintertime eukaryotic community from sea ice and open water in the Baltic Sea, based on sequencing of the 18S rRNA gene. Polar Biol.

[199] Edgcomb VP, Beaudoin D, Gast R, Biddle JF, Teske A (2011). Marine subsurface eukaryotes: the fungal majority. Environ Microbiol.

[200] Monchy S, Sanciu G, Jobard M, Rasconi S, Gerphagnon M (2011). Exploring and quantifying fungal diversity in freshwater lake ecosystems using rdna cloning/sequencing and SSU tag pyrosequencing. Environ Microbiol.

[201] Jobard M, Rasconi S, Solinhac L, Cauchie HM, Sime-Ngando T (2012). Molecular and morphological diversity of fungi and the associated functions in three european nearby lakes. Environ Microbiol.

[202] Genitsaris S, Kormas KA, Moustaka-Gouni M (2009). Microscopic eukaryotes living in a dying lake (lake koronia, greece). FEMS Microbiol Ecol.

[203] Wyngaert S, Rojas-Jimenez K, Seto K, Kagami M, Grossart HP (2018). Diversity and hidden host specificity of chytrids infecting colonial volvocacean algae. J Eukaryot Microbiol.

[204] Rad-Menéndez C, Gerphagnon M, Garvetto A, Arce P, Badis Y (2018). Rediscovering *Zygorhizidium affluens* Canter: molecular taxonomy, infectious cycle, and cryopreservation of a chytrid infecting the bloom-forming diatom *Asterionella formosa*. Appl Environ Microbiol.

[205] Stoeck T, Taylor GT, Epstein SS (2003). Novel eukaryotes from the permanently anoxic Cariaco Basin (Caribbean Sea). Appl Environ Microbiol.

[206] Karpov SA, Kobseva AA, Mamkaeva MA, Mamkaeva KA, Mikhailov KV (2014). *Gromochytrium mamkaevae* gen. & sp. nov. and two new orders: *Gromochytriales* and *Mesochytriales* (*Chytridiomycetes*). Persoonia.

[207] James TY, Letcher PM, Longcore JE, Mozley-Standridge SE, Porter D (2006). A molecular phylogeny of the flagellated fungi (*Chytridiomycota*) and description of a new phylum (*Blastocladiomycota*). Mycologia.

[208] Hussels C (2002). Molekulare Charakterisierung pilzlicher Lebensgemeinschaften in Ackerboeden.

[209] Kupper FC, Maier I, Muller DG, Loiseaux-de Goer S, Guillou L (2006). Phylogenetic affinities of two eukaryotic pathogens of marine macroalgae, *Eurychasma dicksonii* and *Chytridium polysiphoniae*. Cryptogamie Algol.

[210] Nawrocki EP (2009). Structural RNA homology search and alignment using covariance models. https://www.proquest.com/docview/305017419.

[211] Capella-Gutiérrez S, Silla-Martínez JM, Gabaldón T (2009). trimAl: a tool for automated alignment trimming in large-scale phylogenetic analyses. Bioinformatics.

[212] Emms DM, Kelly S (2019). OrthoFinder: phylogenetic orthology inference for comparative genomics. Genome Biol.

[213] Kolde R (2018). pheatmap: Pretty Heatmaps. https://github.com/raivokolde/pheatmap.

[214] Onyshchenko A, Roberts WR, Ruck EC, Lewis JA, Alverson AJ (2021). The genome of a nonphotosynthetic diatom provides insights into the metabolic shift to heterotrophy and constraints on the loss of photosynthesis. New Phytol.

[215] Longcore JE, Qin S, Simmons DR, James TY (2020). *Quaeritorhiza haematococci* is a new species of parasitic chytrid of the commercially grown alga, *Haematococcus pluvialis*. Mycologia.

[216] Simmons DR, Bonds AE, Castillo BT, Clemons RA, Glasco AD (2020). The Collection of Zoosporic Eufungi at the University of Michigan (CZEUM): introducing a new repository of barcoded Chytridiomyceta and Blastocladiomycota cultures. IMA Fungus.

[217] Chung M-H, Lee K-S (2008). Species composition of the epiphytic diatoms on the leaf tissues of three *Zostera* species distributed on the southern coast of Korea. Algae.

[218] Lebreton B, Richard P, Radenac G, Bordes M, Bréret M (2009). Are epiphytes a significant component of intertidal *Zostera noltii* beds?. Aquat Bot.

[219] Atmaja PSP, Bengen DG, Madduppa HH (2021). The second skin of seagrass leaves: a comparison of microalgae epiphytic communities between two different species across two seagrass meadows in Lesser Sunda Islands. Trop Life Sci Res.

[220] Pinckney JL, Micheli F (1998). Microalgae on seagrass mimics: does epiphyte community structure differ from live seagrasses?. J Exp Mar Biol Ecol.

[221] Cox TE, Cebrian J, Tabor M, West L, Krause JW (2020). Do diatoms dominate benthic production in shallow systems? A case study from a mixed seagrass bed. Limnol Oceanogr Letters.

[222] Moncreiff CA, Sullivan MJ, Daehnick AE (1992). Primary production dynamics in seagrass beds of Mississippi Sound: the contributions of seagrass epiphytic algae, sand microflora, and phytoplankton. Mar Ecol Prog Ser.

[223] Coleman VL, Burkholder JM (1994). Community structure and productivity of epiphytic microalgae on eelgrass (*Zostera marina* L.) under water-column nitrate enrichment. J Exp Mar Bio Ecol.

[224] Hasegawa N, Hori M, Mukai H (2007). Seasonal shifts in seagrass bed primary producers in a cold-temperate estuary: dynamics of eelgrass *Zostera marina* and associated epiphytic algae. Aquat Bot.

[225] Frenken T, Alacid E, Berger SA, Bourne EC, Gerphagnon M (2017). Integrating chytrid fungal parasites into plankton ecology: research gaps and needs. Environ Microbiol.

[226] Wawra S, Fesel P, Widmer H, Timm M, Seibel J (2016). The fungal-specific β-glucan-binding lectin FGB1 alters cell-wall composition and suppresses glucan-triggered immunity in plants. Nat Commun.

[227] Martin F, Aerts A, Ahrén D, Brun A, Danchin EGJ (2008). The genome of Laccaria bicolor provides insights into mycorrhizal symbiosis. Nature.

[228] Plett JM, Kemppainen M, Kale SD, Kohler A, Legué V (2011). A secreted effector protein of Laccaria bicolor is required for symbiosis development. Curr Biol.

[229] Voß S, Betz R, Heidt S, Corradi N, Requena N (2018). RiCRN1, a crinkler effector from the arbuscular mycorrhizal fungus *Rhizophagus irregularis*, functions in arbuscule development. Front Microbiol.

[230] Koeck M, Hardham AR, Dodds PN (2011). The role of effectors of biotrophic and hemibiotrophic fungi in infection. Cell Microbiol.

[231] Tanaka S, Kahmann R (2021). Cell wall-associated effectors of plant-colonizing fungi. Mycologia.

[232] Thekkiniath J, Zabet-Moghaddam M, Kottapalli KR, Pasham MR, San Francisco S (2015). Quantitative proteomics of an amphibian pathogen, *Batrachochytrium dendrobatidis*, following exposure to thyroid hormone. PLoS One.

[233] Ellison AR, DiRenzo GV, McDonald CA, Lips KR, Zamudio KR (2017). First in vivo *Batrachochytrium dendrobatidis* transcriptomes reveal mechanisms of host exploitation, host-specific gene expression, and expressed genotype shifts. G3 (Bethesda).

[234] van de Vossenberg BTLH, Prodhomme C, van Arkel G, van Gent-Pelzer MPE, Bergervoet M (2019). The *Synchytrium endobioticum* AvrSen1 triggers a hypersensitive response in *Sen1* potatoes while natural variants evade detection. Mol Plant Microbe Interact.

[235] Glass NL, Schmoll M, Cate JHD, Coradetti S (2013). Plant cell wall deconstruction by ascomycete fungi. Annu Rev Microbiol.

[236] Zhao Z, Liu H, Wang C, Xu JR (2013). Comparative analysis of fungal genomes reveals different plant cell wall degrading capacity in fungi. BMC Genomics.

[237] Ospina-Giraldo MD, Griffith JG, Laird EW, Mingora C (2010). The CAZyome of *Phytophthora* spp.: a comprehensive analysis of the gene complement coding for carbohydrate-active enzymes in species of the genus Phytophthora. BMC Genomics.

[238] Rodriguez‐Moreno L, Ebert MK, Bolton MD, Thomma BPHJ (2018). Tools of the crook‐ infection strategies of fungal plant pathogens. Plant J.

[239] Kubicek CP, Starr TL, Glass NL (2014). Plant cell wall–degrading enzymes and their secretion in plant-pathogenic fungi. Annu Rev Phytopathol.

[240] Lu H, Wei T, Lou H, Shu X, Chen Q (2021). A critical review on communication mechanism within plant-endophytic fungi interactions to cope with biotic and abiotic stresses. J Fungi (Basel).

[241] De Wit PJGM, Mehrabi R, Van den Burg HA, Stergiopoulos I (2009). Fungal effector proteins: past, present and future. Mol Plant Pathol.

[242] Lange L, Barrett K, Pilgaard B, Gleason F, Tsang A (2019). Enzymes of early-diverging, zoosporic fungi. Appl Microbiol Biotechnol.

[243] Rosling A, Eshghi Sahraei S, Kalsoom Khan F, Desirò A, Bryson AE (2024). Evolutionary history of arbuscular mycorrhizal fungi and genomic signatures of obligate symbiosis. BMC Genomics.

[244] Chang Y, Desirò A, Na H, Sandor L, Lipzen A (2019). Phylogenomics of endogonaceae and evolution of mycorrhizas within mucoromycota. New Phytologist.

[245] Tisserant E, Malbreil M, Kuo A, Kohler A, Symeonidi A (2013). Genome of an arbuscular mycorrhizal fungus provides insight into the oldest plant symbiosis. Proc Natl Acad Sci USA.

[246] Newell SY, Fell JW (1980). Mycoflora of Turtlegrass (*Thalassia testudinum* König) as recorded after seawater incubation. Bot Mar.

[247] Poulin R, Krasnov BR, Mouillot D (2011). Host specificity in phylogenetic and geographic space. Trends Parasitol.

[248] Van den Wyngaert S, Ganzert L, Seto K, Rojas-Jimenez K, Agha R (2022). Seasonality of parasitic and saprotrophic zoosporic fungi: linking sequence data to ecological traits. ISME J.

[249] Kagami M, Seto K, Nozaki D, Nakamura T, Wakana H (2021). Single dominant diatom can host diverse parasitic fungi with different degree of host specificity. Limnol Oceanogr.

[250] Sassenhagen I, Irion S, Jardillier L, Moreira D, Christaki U (2020). Protist interactions and community structure during early autumn in the kerguelen region (southern ocean). Protist.

[251] Sassenhagen I, Langenheder S, Lindström ES (2023). Infection strategies of different chytrids in a diatom spring bloom. Freshw Biol.

[252] Haraldsson M, Gerphagnon M, Bazin P, Colombet J, Tecchio S (2018). Microbial parasites make cyanobacteria blooms less of a trophic dead end than commonly assumed. ISME J.

[253] Garvetto A, Badis Y, Perrineau M-M, Rad-Menéndez C, Bresnan E (2019). Chytrid infecting the bloom-forming marine diatom *Skeletonema* sp.: morphology, phylogeny and distribution of a novel species within the Rhizophydiales. Fungal Biol.

[254] Fernández-Valero AD, Reñé A, Timoneda N, Sampedro N, Garcés E (2022). Dinoflagellate hosts determine the community structure of marine Chytridiomycota: demonstration of their prominent interactions. Environ Microbiol.

[255] McKindles KM, Jorge AN, McKay RM, Davis TW, Bullerjahn GS (2021). Obligate parasite of planktothrix agardhii in a laurentian great lakes embayment. Appl Environ Microbiol.

[256] Seto K, Degawa Y (2015). Cyclopsomyces plurioperculatus: a new genus and species of lobulomycetales (chytridiomycota, chytridiomycetes) from japan. Mycologia.

[257] Hassett BT, Gradinger R (2016). Chytrids dominate arctic marine fungal communities. Environ Microbiol.

[258] Rojas-Jimenez K, Rieck A, Wurzbacher C, Jürgens K, Labrenz M (2019). A salinity threshold separating fungal communities in the baltic sea. Front Microbiol.

[259] Brubaker SW, Bonham KS, Zanoni I, Kagan JC (2015). Innate immune pattern recognition: a cell biological perspective. Annu Rev Immunol.

[260] Couto D, Zipfel C (2016). Regulation of pattern recognition receptor signalling in plants. Nat Rev Immunol.

[261] Soanes DM, Talbot NJ (2010). Comparative genome analysis reveals an absence of leucine-rich repeat pattern-recognition receptor proteins in the kingdom Fungi. PLoS One.

[262] Daskalov A (2023). Emergence of the fungal immune system. iScience.

[263] Gaspar ML, Pawlowska TE (2022). Innate immunity in fungi: is regulated cell death involved?. PLoS Pathog.

[264] Muehlstein LK, Amon JP, Leffler DL (1988). Chemotaxis in the marine fungus *Rhizophydium littoreum*. Appl Environ Microbiol.

[265] Moss AS, Reddy NS, Dortaj IM, San Francisco MJ (2008). Chemotaxis of the amphibian pathogen *Batrachochytrium dendrobatidis* and its response to a variety of attractants. Mycologia.

[266] Scholz B, Küpper FC, Vyverman W, Ólafsson HG, Karsten U (2017). Chytridiomycosis of marine diatoms—the role of stress physiology and resistance in parasite-host recognition and accumulation of defense molecules. Mar Drugs.

[267] Pfeifer L, van Erven G, Sinclair EA, Duarte CM, Kabel MA (2022). Profiling the cell walls of seagrasses from A (*Amphibolis*) to Z (*Zostera*). BMC Plant Biol.

[268] Wang Y, Verbrugghe E, Meuris L, Chiers K, Kelly M (2021). Epidermal galactose spurs chytrid virulence and predicts amphibian colonization. Nat Commun.

[269] Rovenich H, Boshoven JC, Thomma BP (2014). Filamentous pathogen effector functions: of pathogens, hosts and microbiomes. Curr Opin Plant Biol.

[270] Sánchez-Vallet A, Mesters JR, Thomma BPHJ (2015). The battle for chitin recognition in plant-microbe interactions. FEMS Microbiol Rev.

[271] Cao C, Xue C (2021). More than just cleaning: ubiquitin-mediated proteolysis in fungal pathogenesis. Front Cell Infect Microbiol.

[272] Finley D, Ulrich HD, Sommer T, Kaiser P (2012). The ubiquitin-proteasome system of *Saccharomyces cerevisiae*. Genetics.

[273] Liu TB, Xue C (2011). The ubiquitin-proteasome system and F-box proteins in pathogenic fungi. Mycobiology.

[274] Pesin JA, Orr-Weaver TL (2008). Regulation of APC/C activators in mitosis and meiosis. Annu Rev Cell Dev Biol.

[275] Rotin D, Kumar S (2009). Physiological functions of the HECT family of ubiquitin ligases. Nat Rev Mol Cell Biol.

[276] Grifoll-Romero L, Pascual S, Aragunde H, Biarnés X, Planas A (2018). Chitin deacetylases: structures, specificities, and biotech applications. Polymers (Basel).

[277] Khokhani D, Carrera Carriel C, Vayla S, Irving TB, Stonoha-Arther C (2021). Deciphering the chitin code in plant symbiosis, defense, and microbial networks. Annu Rev Microbiol.

[278] Oldroyd GED (2013). Speak, friend, and enter: signalling systems that promote beneficial symbiotic associations in plants. Nat Rev Microbiol.

[279] Aspeborg H, Schrader J, Coutinho PM, Stam M, Kallas A (2005). Carbohydrate-active enzymes involved in the secondary cell wall biogenesis in hybrid aspen. Plant Physiol.

[280] Beauvais A, Latgé J-P (2018). Special issue: fungal cell wall. *J Fungi (Basel*).

[281] Henrissat B, Davies G (1997). Structural and sequence-based classification of glycoside hydrolases. Curr Opin Struct Biol.

[282] Ökmen B, Bachmann D, de Wit PJGM (2019). A conserved GH17 glycosyl hydrolase from plant pathogenic Dothideomycetes releases a DAMP causing cell death in tomato. Mol Plant Pathol.

[283] Chrismas N, Cunliffe M (2020). Depth-dependent mycoplankton glycoside hydrolase gene activity in the open ocean—evidence from the Tara Oceans eukaryote metatranscriptomes. ISME J.

[284] Cunliffe M, Hollingsworth A, Bain C, Sharma V, Taylor JD (2017). Algal polysaccharide utilisation by saprotrophic planktonic marine fungi. Fungal Ecol.

[285] Becker S, Tebben J, Coffinet S, Wiltshire K, Iversen MH (2020). Laminarin is a major molecule in the marine carbon cycle. Proc Natl Acad Sci USA.

[286] Abramyan J, Stajich JE (2012). Species-specific chitin-binding module 18 expansion in the amphibian pathogen *Batrachochytrium dendrobatidis*. mBio.

[287] Powell AJ, Conant GC, Brown DE, Carbone I, Dean RA (2008). Altered patterns of gene duplication and differential gene gain and loss in fungal pathogens. BMC Genomics.

[288] Sexton AC, Howlett BJ (2006). Parallels in fungal pathogenesis on plant and animal hosts. *Eukaryot Cell*.

[289] Rappleye CA, Goldman WE (2006). Defining virulence genes in the dimorphic fungi. Annu Rev Microbiol.

[290] Halaouli S, Asther M, Sigoillot J-C, Hamdi M, Lomascolo A (2006). Fungal tyrosinases: new prospects in molecular characteristics, bioengineering and biotechnological applications. J Appl Microbiol.

[291] Camejo D, Guzmán-Cedeño Á, Moreno A (2016). Reactive oxygen species, essential molecules, during plant-pathogen interactions. Plant Physiol Biochem.

[292] Perlin MH, Andrews J, San Toh S. (2014). Essential Letters in the Fungal Alphabet: ABC and MFS Transporters and Their Roles in Survival and Pathogenicity. Advances in Genetics.

