# Supplementary material for: Eukaryotic metagenome-assembled genomes recovered from deep metagenomic sequencing of the seagrass, Zostera marina, include a novel chytrid in the order Lobulomycetales
Source: Microb Genom. 2026 Sep 23;12(9):001845. doi: 10.1099/mgen.0.001845 (PMC13618815; doi:10.1099/mgen.0.001845)

Supplementary Materials for:

Eukaryotic MAGs recovered from deep metagenomic sequencing of the seagrass, *Zostera marina,* include a novel chytrid in the order Lobulomycetales

Cassandra L. Ettinger[1,#], Jonathan A. Eisen[2,3,4], Jason E. Stajich[1,5]

[1]Department of Microbiology and Plant Pathology, University of California, Riverside, CA, United States
[2]Genome Center, University of California, Davis, CA, United States
[3]Department of Evolution and Ecology, University of California, Davis, CA, United States
[4]Department of Medical Microbiology and Immunology, University of California, Davis, Davis, CA, United States
[5]Institute for Integrative Genome Biology, University of California, Riverside, Riverside, CA, United States

[#]**Corresponding Author:** Cassandra L. Ettinger, clettinger@ucdavis.edu

Keywords: *Zostera marina*, marine fungi, microbial eukaryotes, eelgrass, metagenomics, genome assembly, genome annotation, Chytridiomycota, Bacillariophyta, Prymnesiophyceae, Lobulomycetales

Supplemental Table Legends and Figures:

Table S1. Genomes used in phylogenetic analysis of gyrista. Here we provide details on each genome obtained from NCBI including assembly statistics, BioProject number, assembly accession number, and appropriate data citation.

Table S2. Genomes used in phylogenetic and orthogroup analyses of fungi. Here we provide details on each genome obtained from NCBI including taxonomic information, assembly statistics, BioProject number, and assembly accession number. We also report the predicted trophic mode of each organism and appropriate data citation.

Table S3. Genomes used in phylogenetic analysis of haptista. Here we provide details on each genome obtained from NCBI or METdb including BUSCO statistics, assembly accession number, and appropriate data citation.

Table S4. Sequences used in phylogenetic analysis of SGEUK-03 18S ribosomal RNA gene. Here we provide details on each sequence obtained from NCBI including taxonomic clade, species and strain ID, as well as NCBI accession number, and appropriate data citation.

Table S5. CAZyme distribution of SGEUK-03. Here we provide the counts of annotated genes with CAZyme domain hits, including the CAZyme category, CAZyme domain and gene count.

Figure S1. Effector and CAZyme profiles for SGEUK-03. (A) Bar plot depicting the number of secreted genes in the SGEUK-03 genome that are predicted to be plant apoplastic and cytoplasmic effectors. (B) Bar plot depicting the frequency distribution of predicted CAZymes in the SGEUK-03 genome across CAZyme families.

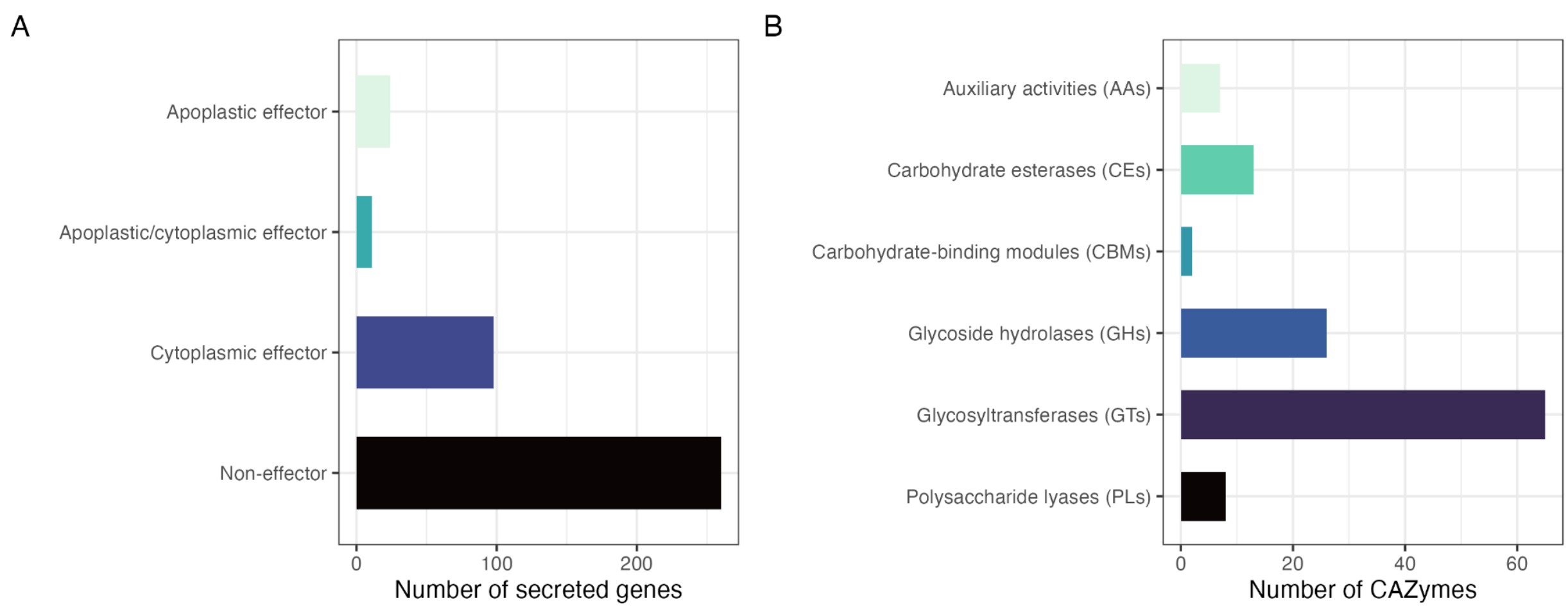

Supplement: Supplementary file 1 [file mgen-12-01845-s001.pdf]
